# Viscoelastometric Testing to Assess Hemostasis of COVID-19: A Systematic Review

**DOI:** 10.3390/jcm10081740

**Published:** 2021-04-16

**Authors:** Marion Bareille, Michaël Hardy, Jonathan Douxfils, Stéphanie Roullet, Dominique Lasne, Jerrold H. Levy, Alain Stépanian, Sophie Susen, Corinne Frère, Thomas Lecompte, François Mullier

**Affiliations:** 1 Namur Thrombosis and Hemostasis Center (NTHC), CHU UCL Namur, Université Catholique de Louvain, 5530 Yvoir, Belgium; francois.mullier@uclouvain.be; 2 Service D’anesthésiologie, CHU UCL Namur, Université Catholique de Louvain, 5530 Yvoir, Belgium; michael.hardy@uclouvain.be; 3 Namur Thrombosis and Hemostasis Center (NTHC), Département de Pharmacie, Université de Namur, 5000 Namur, Belgium; jonathan.douxfils@unamur.be; 4 Qualiblood S.A., 5000 Namur, Belgium; 5 CHU Bordeaux, Service D’Anesthésie-Réanimation Tripode, 33000 Bordeaux, France; stephanie.roullet@chu-bordeaux.fr; 6 Biologie des Maladies Cardiovasculaire, University Bordeaux, INSERM U1034, 33600 Pessac, France; 7 Laboratoire D’hématologie Générale, Hôpital Universitaire Necker-Enfants Malades, AP-HP, 75015 Paris, France; dominique.lasne@aphp.fr; 8 Departments of Anesthesiology, Critical Care, and Surgery (Cardiothoracic), Duke University School of Medicine, Durham, NC 27710, USA; jerrold.levy@duke.edu; 9 Hôpital Lariboisière, Service D’Hématologie Biologique, Institut de Recherche Saint-Louis, Université de Paris, AP-HP Nord-Université de Paris, EA 3518, 75010 Paris, France; alain.stepanian@aphp.fr; 10 Laboratoire D’Hématologie-Hémostase, Université de Lille, CHU Lille, 59037 Lille, France; sophie.susen@chru-lille.fr; 11 Department of Hematology, Pitié-Salpêtrière Hospital, Assistance Publique Hôpitaux de Paris, INSERM UMRS_1166, Sorbonne Université, 75013 Paris, France; corinne.frere@aphp.fr; 12 Départements de Médecine, Service D’angiologie et D’hémostase et Faculté de Médecine Geneva Platelet Group (GpG), Université de Genève et Hôpitaux Universitaires de Genève, 1205 Genève, Switzerland; thomaspierre.lecompte@hcuge.ch

**Keywords:** viscoelastic test, thromboelastometry, thromboelastography, sonorheometry, ROTEM, TEG, Quantra, ClotPro, coronavirus disease 2019, COVID-19, severe acute respiratory syndrome coronavirus 2, SARS-CoV-2

## Abstract

Infection by SARS-CoV-2 is associated with a high risk of thrombosis. The laboratory documentation of hypercoagulability and impaired fibrinolysis remains a challenge. Our aim was to assess the potential usefulness of viscoelastometric testing (VET) to predict thrombotic events in COVID-19 patients according to the literature. We also (i) analyzed the impact of anticoagulation and the methods used to neutralize heparin, (ii) analyzed whether maximal clot mechanical strength brings more information than Clauss fibrinogen, and (iii) critically scrutinized the diagnosis of hypofibrinolysis. We performed a systematic search in PubMed and Scopus databases until 31st December 2020. VET methods and parameters, and patients’ features and outcomes were extracted. VET was performed for 1063 patients (893 intensive care unit (ICU) and 170 non-ICU, 44 studies). There was extensive heterogeneity concerning study design, VET device used (ROTEM, TEG, Quantra and ClotPro) and reagents (with non-systematic use of heparin neutralization), timing of assay, and definition of hypercoagulable state. Notably, only 4 out of 25 studies using ROTEM reported data with heparinase (HEPTEM). The common findings were increased clot mechanical strength mainly due to excessive fibrinogen component and impaired to absent fibrinolysis, more conspicuous in the presence of an added plasminogen activator. Only 4 studies out of the 16 that addressed the point found an association of VETs with thrombotic events. So-called functional fibrinogen assessed by VETs showed a variable correlation with Clauss fibrinogen. Abnormal VET pattern, often evidenced despite standard prophylactic anticoagulation, tended to normalize after increased dosing. VET studies reported heterogeneity, and small sample sizes do not support an association between the poorly defined prothrombotic phenotype of COVID-19 and thrombotic events.

## 1. Introduction

In contrast to conventional clotting tests, viscoelastic tests (VETs) monitor changes of viscoelastic properties of a forming and evolving clot from whole blood, before and beyond the clotting point; they are often referred to as a global hemostasis test, although some aspects of hemostasis are not explored [1,2,3]. Coagulation occurs in the presence of platelets and red blood cells, and fibrinolysis can translate into a decrease in clot mechanical strength after its maximum has been reached, but clot retraction seems to play a role here as well [4,5,6]. VETs are based on the mechanical properties of the clot, like mechanical strength, and are influenced by its composition in platelets, fibrin, red blood cells, and factor XIII [7,8,9]. To our knowledge, the assessment of factor XIII by VETs has not been investigated in COVID-19 patients.

VETs have been considered to provide a comprehensive assessment of the dynamic process of blood clot formation and subsequent lysis. As they can be performed bedside as point-of-care testing and can give useable results about clot formation and a potential hyperfibrinolysis within one hour, they are chiefly considered as convenient tools for real-time assessment of coagulation and fibrinolysis in whole blood and have been gaining in popularity in various hemorrhagic situations, such as cardiac surgery, obstetrics, and traumatology over decades, for the management of acutely bleeding patients [3]. By contrast, COVID-19 disturbance of hemostasis is likely a combination of hypercoagulability and impaired fibrinolysis (a prothrombotic laboratory phenotype), at least in part, contributing to the thrombotic risk and the prothrombotic laboratory phenotype, but VETs have been nevertheless suggested to be potentially useful, in line with previous works on sepsis [10] and trauma [11,12], for example.

Of note, VETs share the same limitations as all currently available clinical lab tests, i.e., negligible effect of endogenous anticoagulants, absence of endothelium, and very low shear in a close system. Furthermore, there are good reasons to challenge the interpretation of hypercoagulability and to question the ability to sensitively detect and accurately quantify hypofibrinolysis, especially when a value equal to zero belongs to the manufacturer’s reference range.

Our aim was to assess the potential clinical usefulness of VETs to predict clinical outcomes (mainly thrombotic events) in COVID-19 patients through this systematic review. We also (i) analyzed the impact of anticoagulation and the methods used to neutralize heparin (in other words, was heparin duly neutralized?), (ii) disentangled reported alterations in clotting dynamics and analyzed whether maximal clot mechanical strength brings more information than Clauss fibrinogen, and (iii) critically scrutinized the documentation of hypofibrinolysis with VET under various reactive conditions. The term ‘hypercoagulable state’ will be uniformly used to refer to the investigators’ interpretation of VET findings; we will discuss to what extent this is an appropriate interpretation.

The preanalytical aspects, which are crucial in laboratory hemostasis but scarcely mentioned among the retrieved studies, are beyond the scope of this review and will not be addressed.

## 2. Materials and Methods

### 2.1. Search Methodology

We performed a systematic literature search in PubMed and Scopus databases, regardless of publication status, using the following keywords ‘viscoelastic test OR thromboelastometry OR thromboelastography OR sonorheometry OR ROTEM OR TEG OR Quantra OR ClotPro’ AND ‘coronavirus disease 2019 OR COVID-19 OR severe acute respiratory syndrome coronavirus 2 OR SARS-CoV-2’. Search strategy is provided as Data S1. We also searched the reference lists of selected articles for additional relevant works, and we did not restrict our search to articles published in English and found some articles in Russian and Hungarian. In addition, reviewers performed manual searches and cross-references in the retrieved papers. The last search was conducted on 31 December 2020. Our review followed the PRISMA (Preferred Reporting Items for Systematic Reviews and Meta-analysis) guidelines [13], and the PRISMA summary table can be found as Data S2. Due to a considerable heterogeneity among the retrieved studies, we did not extend our systematic review to a meta-analysis.

### 2.2. Study Selection

All references retrieved from our search were screened based upon their title and abstract to assess eligibility. If they were considered relevant, the full-text articles were analyzed to check if they met the selection criteria as follows. As COVID-19 pandemic is a recent phenomenon, and due to the relatively small number of published data on VETs, we did not restrict eligibility according to patients’ characteristics, disease severity, or treatment modalities. Studies of any design and case reports, including original data from VETs in COVID-19 patients with neither pregnancy nor known history of coagulation disorder, were deemed eligible. All relevant studies regardless of methodological quality were included when the full-text article was available (Table 1).

Reviews, position articles, and guidelines were excluded. All kind of VETs were included but were analyzed separately.

### 2.3. Data Extraction

For each study, data regarding author identification, geographic location, study design, number of patients and their characteristics (including comorbidities and thrombotic events), prospective design or not, timing of blood collection and anticoagulation status, type of VET device used and results, and the results of other conventional hemostasis tests (platelet count, fibrinogen and D-dimers plasma levels), and C-reactive protein were extracted with the aid of a systematic chart.

### 2.4. A Concise Overview of the Different VET Devices

Viscoelastometric testing (VET) should be performed either immediately with native whole blood or within four hours after drawing if performed with whole citrated blood, as most often done [1,3].

ROTEM devices and TEG5000 all rely on the movement of a pin and a cup relative to each other; in the former, the cuvette is fixed, and the pin oscillates, and vice versa in the latter. The oscillations are recorded and graphically displayed with the characteristic normal tuning fork shape [3]. The conventional clotting point roughly corresponds to the reaction time R for TEG, and to the clotting time CT for ROTEM, ClotPro, and Quantra; extended fibrin polymerization is monitored with the kinetics time K and α angle for TEG and with CFT and α angle for ROTEM and ClotPro; the eventual result is maximal mechanical strength (maximal amplitude MA for TEG, maximal clot firmness MCF for ROTEM and ClotPro and clot stiffness CS for Quantra) and its subsequent decrease, as a result of ‘endogenous’ fibrinolysis monitored by lysis of the clot at given time x LY(x) for TEG and by maximal lysis ML or lysis of the clot at a given time x (LI(x)) for ROTEM and ClotPro, at least in part [2,3,14].

Coagulation can be initiated through the contact phase or the tissue factor pathway (often referred to as intrinsic or extrinsic pathways, respectively) and needs recalcification when citrated blood is used [3]. If the nature of the initiating agents is known, their concentrations are not disclosed. Regarding the former pathway, the limitations of aPTT testing apply, although ‘clotting times’ are longer, suggesting a lower amount of contact phase activator (kaolin, celite, or ellagic acid) and higher calcium concentration. The different well-known behaviors of those reagents in case of defective contact phase, abnormal factor VIII levels, high CRP (C-reactive protein) levels, lupus anticoagulant, or heparin must be borne in mind. Two reagents can be used to neutralize heparin, either polybrene (hexadimethrine bromide) or heparinase; two to inhibit the platelet contribution to mechanical clot properties, namely cytochalasin D and abciximab, sometimes both together; lastly, two to inhibit fibrinolysis, either aprotinin or tranexamic acid [3]. To what extent those inhibitions are fully achieved is not entirely clear.

#### 2.4.1. ROTEM

Three versions of the ROTEM device exist: from the oldest to the most recent, ROTEM-gamma, ROTEM-delta, and the brand-new version ROTEM-sigma. The main difference between them is that ROTEM-gamma and -delta need manual pipetting of the blood sample and the reagents into cups, whereas ROTEM-sigma is a completely automated, closed system. For the latter, reagents consist of a consumable ready-to-use cartridge with four parallel channels prefilled with specific lyophilized reagents [15]. All ROTEM versions can perform the same assays, namely INTEM, HEPTEM, EXTEM, FIBTEM, and APTEM, to investigate the intrinsic pathway (with and without heparinase), the extrinsic pathway, the fibrinogen component, and the fibrinolysis with aprotinin, respectively. Of note, EXTEM, FIBTEM, and APTEM reagents contain polybrene and HEPTEM contain heparinase to neutralize heparin (Table A1) [2,16]. They report the same parameters: clotting time (CT), clot formation time (CFT), α angle, “amplitude of the clot” at a given time x (A(x)), maximum clot firmness (MCF), clot lysis index (LI(x)), and maximum lysis (ML) (Table A2).

#### 2.4.2. TEG

Briefly, regarding TEG5000 a blood sample is pipetted into a cup; liquid reagents are added; ultimately, a fixed pin connected to a detector system is then put in the cup. The graphical representation is called TEMogram. TEG6s for its part is a completely closed and automated system. In contrast to its predecessor TEG5000, it relies on sonorheometry. Reagents consist of a consumable, ready-to-use cartridge with four parallel channels prefilled with specific lyophilized reagents (Table A3) [17,18].

The two versions of the TEG device can perform the same assays, namely Kaolin TEG with (CKH) or without heparinase (CK), RapidTEG (CRT), and TEG Functional Fibrinogen (CFF), and offer the same parameters: reaction time (R), kinetics time (K), α angle, maximum amplitude (MA), and fibrinolytic activity (Ly) [3,19]. Of note, heparin neutralization differs between TEG500, where neutralization can occur in virtually any channel by using heparinase-coated cups, and TEG6s, where neutralization occurs only in the CKH channel thanks to heparinase (Table A4) [3].

#### 2.4.3. Quantra

The Quantra device also uses sonorheometry. Briefly, an acoustic radiation force is applied to the blood sample. As the blood clot forms, it starts to resonate: oscillations are then correlated with the shear modulus of the blood sample. The resistance of the sample to shear forces can be quantified by the time delay between the ultrasound pulse emission and the returning echoes [20,21,22].

Reagents consist of a consumable, ready-to-use cartridge with four parallel channels prefilled with specific lyophilized reagents [22]. There are currently two kinds of cartridges: the QPlus cartridge and the QStat one dedicated to exploring fibrinolysis [23]. Measurements of clot coagulation time with (CTH) or without (CT) heparinase and coagulation initiation with kaolin, clot stiffness (CS) after initiation with thromboplastin, and fibrinogen contribution to the overall clot stiffness (FCS) after platelet inhibition with abciximab are performed simultaneously in four parallel channels. Of note, channel 2 contains heparinase, and channels 3 and 4 contain polybrene to neutralize heparin. Platelet contribution to clot stiffness (PCS) results from the difference between total CS and FCS (Table A5 and Table A6).

#### 2.4.4. ClotPro

The ClotPro device uses rotational technology similar to ROTEM® (Werfen, Barcelona, Spain), but some differences exist between the two devices. First, in contrast with ROTEM, the cuvette rotates and the pin is stationary [24,25]. Second, reagents for each assay are present in dry form in a sponge located in the pipette tip; during pipetting of the patient sample, the reagent is automatically added to the blood [25]. This device can perform the same kind of assays as the ROTEM device (EX-test, IN-test, HI-test, FIB-test, AP-test) plus some other specific ones (RVV-test, ECA-test), and offer similar parameters. Of note, EX-test, tPA-test, and FIB-test contain polybrene to neutralize heparin (Table A7 and Table A8) [24].

## 3. Results

### 3.1. Literature Search

**Figure 1:**
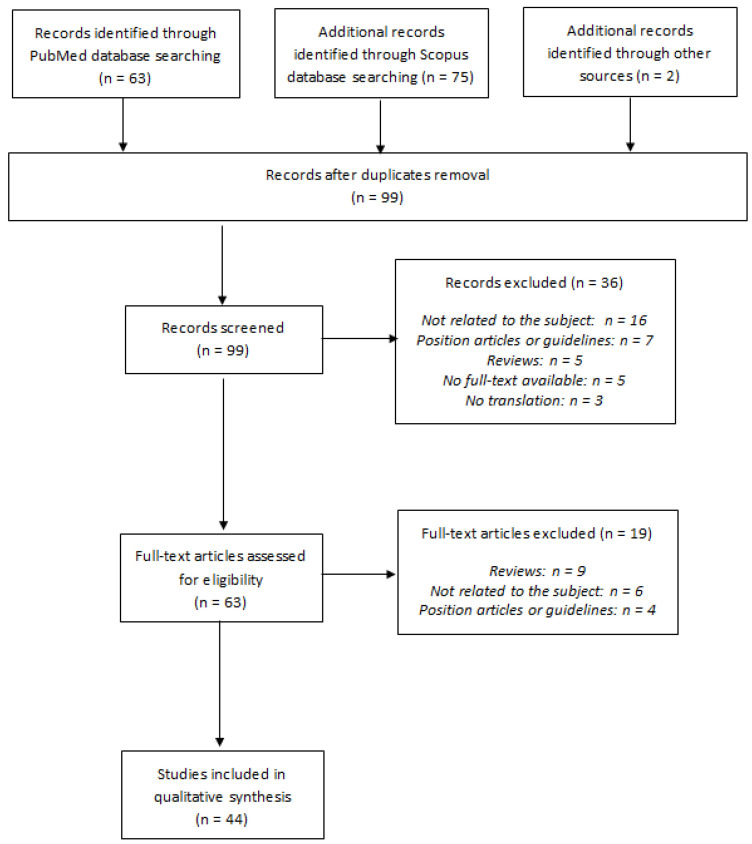
Literature search and selection flow chart according to PRISMA statement.

Our literature search and selection flow chart according to PRISMA statement [13] is summarized in Figure 1.

We identified 140 references, resulting in 97 unique citations after duplicates removal. Two additional articles were identified through other sources. Each title and abstract were screened, and 36 references were excluded either because they were not related to the subject (*n* = 16), because they were position articles or guidelines (*n* = 7) or reviews (*n* = 5), or because there was no full-text available at this time (*n* = 5) or no possible translation (*n* = 5). A total of 63 potentially eligible articles were considered for inclusion, and the full-text articles were retrieved. The most common reasons for exclusion after the full-text evaluation were that papers were reviews (*n* = 19), not related to the subject (*n* = 6), or position articles or guidelines (*n* = 4). Finally, 44 references [24,26,27,28,29,30,31,32,33,34,35,36,37,38,39,40,41,42,43,44,45,46,47,48,49,50,51,52,53,54,55,56,57,58,59,60,61,62,63,64,65,66,67,68] met the eligibility criteria.

### 3.2. Originality of Our Systematic Review as Compared to the Existing Ones on the Subject

Reviews have already been published recently, two of them only being systematic [69,70,71,72], but none has so far investigated the four major commercially available VET devices (i.e., ROTEM, TEG, ClotPro, and Quantra) or included such a large number of studies (*n* = 44). Characteristics of each review are summarized in Table 2.

Overall, case reports were excluded (except for one systematic review [71]); few studies were available and presented extensive heterogeneity.

### 3.3. Characteristics of the Selected Studies

Quality assessment of the selected study was performed using the Scottish Intercollegiate Guidelines Network (SIGN) grading system [73]. Overall, the retrieved studies were of low (3, “non analytic studies”) to moderate quality (2+, “well-conducted case control or cohort studies with a low risk of confounding or bias and a moderate probability that the relationship is causal”), and details can be found as Data S3. Characteristics of the selected studies are summarized in Table 3.

A total of 1538 inpatients were studied, of which 1393 were COVID-19-positive, among whom 1189 were ICU patients. At least one VET was performed during the hospital stay of 1208 patients, of whom 1063 were COVID-19 patients hospitalized either in an ICU (893 patients) or in a medical ward (IMW, 170 patients). The remaining 145 patients were sex- and age-matched non-COVID-19 controls hospitalized either in the ICU (89 patients) or in IMW (56 patients) for ARDS (acute respiratory distress syndrome) or pneumonia non-related to SARS-CoV-2, or for postoperative care. One article [33] reported data about eight hospitalized children either in a pediatric ward or in a pediatric ICU (PICU).

Among the 44 retrieved studies, 19 were prospective [28,29,30,31,32,39,40,41,42,43,44,45,46,52,59,60,61,66,67], 18 were retrospective [24,27,33,34,35,36,48,49,51,53,54,55,56,57,58,62,63,64], one was a cross-sectional study [47], and six were case reports [26,37,38,50,65,68]. There was no randomized controlled trial (VET versus no VET).

VETs were performed using ROTEM (25 studies), TEG (15 studies), Quantra (two prospective studies [66,67]) and ClotPro (one retrospective study [24] and one case report [68]); no study compared two devices. Among articles reporting data about TEG, four were prospective studies [52,59,60,61], ten were retrospective studies [51,53,54,55,56,57,58,62,63,64], and one was a case report [65]. Among articles dealing with ROTEM, thirteen were prospective studies [28,29,30,31,32,39,40,41,42,43,44,45,46], seven were retrospective studies [27,33,34,35,36,48,49], one was a cross-sectional study [47], and four were case reports [26,37,38,50].

Testing was carried out either on admission or within the following days, but the timing of blood collection for VET was specified only for 29 studies [24,26,27,29,30,31,33,35,36,37,38,41,43,44,45,46,47,48,50,53,55,56,57,58,59,65,66,67]. In some studies, the measurements were repeated during the patient’s stay, either because of a pre-established protocol [26,27,29,45,53,59,60,67] or because of the occurrence of a thromboembolic event [65,68]. Number of VETs performed during a patient’s stay ranged from 1 to 5 [29].

### 3.4. Characteristics of the Included Patients

Characteristics of the included patients are shown in Table 4.

The number of COVID-19 patients with at least one VET performed in each article ranged from 1 [26,38,65] to 64 [58]. Mean or median adult COVID-19 patients ages ranged from 39 [65] to 84 years [68]. Excluding case reports, the proportion of women among the studies reporting gender ranged from 0 [40] to 50% [29,33,36].

Overall, most patients presented with overweight or obesity, associated with other additional co-morbidities such as diabetes or hypertension. Overall, COVID-19 patients were characterized by hyperfibrinogenemia, marked increased D-dimer levels, and increased C-reactive protein (CRP). The majority of patients received thromboprophylaxis either with unfractionated heparin (UFH) or low molecular weight heparin (LMWH) (at usual prophylactic doses or higher) according to published guidance [74,75,76] or local protocols. Thrombotic events (such as deep vein thrombosis, pulmonary embolism, ischemic stroke, or acute kidney injury) were reported as an outcome in 36 articles [24,26,27,28,29,31,33,34,36,37,38,39,40,42,43,44,47,48,49,51,53,54,55,56,57,58,59,60,61,62,63,64,65,66,67,68].

### 3.5. Results of the Viscoelastic Tests

#### 3.5.1. ROTEM

ROTEM devices were used in 25 studies with a total of 708 patients, of whom 435 were ICU COVID-19 patients, most of them intubated and mechanically ventilated. Five studies compared results from COVID-19 patients versus non COVID-19 patients: one reported data from non-ICU patients [46], whereas the four other ones reported data from ICU patients [32,35,36,45]. Six studies reported data from both ICU and IMW COVID-19 patients [28,32,33,39,41,48].

Data from ROTEM gamma, delta, and sigma were reported in one study, thirteen studies [28,29,30,31,32,33,34,35,36,37,38,39,40], and ten [41,42,43,44,45,46,47,48,49,50], respectively. One case report did not specify the device [26]. Results are displayed in Table 5 (EXTEM, INTEM, and FIBTEM assays), Table 6 (INTEM and HEPTEM assays), and Table 7 (EXTEM and TEP-tPA).

As a general rule, three assays were performed, mostly INTEM (19 studies), EXTEM (23 studies), and FIBTEM (23 studies). The great majority of the articles reported results from EXTEM assay with or without INTEM assay and associated with FIBTEM assay. Only four articles [26,41,44,50] reported data from HEPTEM assay (Table 6), while almost all patients received anticoagulation by UFH or LMWH at least at a prophylactic dose. The APTEM assay results were only reported by one case report [26] and were consistent with the absence of hyperfibrinolysis. Two studies reported data from TEM-tPA (Table 7), an investigator-modified assay derived from EXTEM assay to investigate a potential hypofibrinolysis [39,40].

Among the 18 articles reporting data from EXTEM, INTEM, and FIBTEM assays, 16 [26,27,29,31,33,36,38,41,42,44,45,46,47,48,49,50] found an increase in “amplitude of the clot” in the three assays, and 2 only in EXTEM and FIBTEM assays [43], or in FIBTEM assay alone [28]. Among the four articles reporting data from EXTEM and FIBTEM only [30,34,35,37], EXTEM only [32], EXTEM and TEM-tPA only [40], and TEM-tPA only [39], an increased in the “amplitude of the clot” was also a common finding.

Besides the increased clot amplitude, other abnormalities were interpreted as suggesting a hypercoagulable state. First, a shortened CFT in EXTEM, INTEM, FIBTEM, and/or HEPTEM was evidenced in 14 studies [26,27,31,32,33,38,41,42,43,44,45,46,49,50] out of 18, whereas the others found no abnormalities or even a prolonged CFT [28,29,35,37]. Second, four studies [32,38,39,50] out of five showed an increase in α angle in EXTEM or in TEM-tPA, whereas the last reported a normal or even a decrease one [37].

Some articles also reported a reduced or absent fibrinolysis, better evidenced with added plasminogen activator (tissue plasminogen activator, tPA) [39,40] than without [26,30,32,34,37,42,43,44,46,48], whereas others did not report any abnormality [27,31,35,45,46,50]. Few articles studied fibrinolysis over time and found it persistently defective [26,29]. Fibrinolysis was weaker in ICU COVID-19 patients than in non-ICU COVID-19 patients (*p* < 0.05 [32,39,48]), and among ICU patients in those with SOFA score > 10 (*p* = 0.004 [29]) or with thrombotic events (*p* = 0.001 [44]). However, there was no difference between ICU COVID-19 patients and ICU non COVID-19 patients (*p* > 0.05 [32]).

Among the five studies comparing results from COVID-19 patients versus non-COVID-19 (surgical or suffering from pneumonia or ARDS) patients [32,35,36,45,46], three reported a hypercoagulable pattern only in COVID-19 patients (*p* < 0.05 [35,36,45]), a finding which could be explained by a fibrinogen level remaining within the reference range for non-COVID-19 patients [45]. The other two [32,46] showed a similar hypercoagulable pattern in COVID-19 and non-COVID-19 patients as compared with healthy controls (*p* < 0.001), however with a more pronounced one (*p* < 0.05) in COVID-19 patients despite a similar fibrinogen level [46].

Among the five studies reporting data from COVID-19 adult patients both in an ICU and an IMW [28,32,39,41,48], one showed a similar hypercoagulable profile (increased maximum clot firmness) for both groups (*p* > 0.05 [48]), whereas the four others showed a similar hypercoagulable pattern (increased “amplitude of the clot” or maximum clot firmness with or without a decreased CFT) for both groups compared with healthy controls or manufacturer’s reference range; however, the hypercoagulable pattern was more pronounced for ICU patients (*p* < 0.05 between both groups).

Results reported in children [33] showed a hypercoagulable pattern comparable to adults with an increased in MCF in INTEM, EXTEM, and FIBTEM assays and a slightly decreased CFT.

Overall, the authors concluded from those described reports that COVID-19 patients exhibit a hypercoagulable profile characterized by an increased fibrinogen component of clot mechanical strength reflected by an increase in clot amplitude (A(x)) and/or maximum clot firmness (MCF), sometimes associated with a shorter clot formation time CFT [26,27,31,32,33,38,41,42,43,44,45,46,49,50], or an increased α angle [32,38,39,50]. This pattern was often associated with an impaired or absent fibrinolysis [26,30,32,34,37,39,40,42,43,44,46,48].

In summary, four points are worthy of consideration. First, the hypercoagulable profile defined just above was observed early in the clinical course of the disease [41,50]. Second, it was observed in both ICU and non-ICU COVID-19 patients [28,32,39,41,48]. Third, it persisted over time from admission up to 10 to 14 days later [26,27,29,45]. Fourth, it was observed even in the absence of heparin neutralization and despite higher therapeutic intensity anticoagulation administration [26,47]. Of note, only six studies [28,33,34,35,44,49] gathering 195 patients examined the potential association with thrombotic events occurring, and only two [34,44] reported that patients with thromboembolic complications exhibited low or even absent fibrinolysis.

#### 3.5.2. TEG

A total of 403 patients, of whom 402 were COVID-19 ICU patients, had at least one VET performed with TEG. Most of them were intubated and mechanically ventilated. They almost all received anticoagulation by UFH or LMWH, at least at prophylactic dose.

Among the 15 TEG studies, two versions of the device were used: the TEG5000 (*n* = 7) [52,53,54,55,56,57,58] and the brand-new version TEG6s (*n* = 7) [59,60,61,62,63,64,65]. One article reported data without specification of the device [51]. Results are summarized in Table 8.

Kaolin TEG with heparinase (CKH) was the most used assay, as heparin is neutralized, and most patients received heparin. Among the 14 studies using this assay [51,52,53,54,55,56,57,58,59,60,62,63,64,65], an increase in maximum clot amplitude was reported, but this finding needs to be tempered for the following reasons. With patients’ values higher than reference [51,54,57,58,60] or locally established [52,53,56] values, MA was considered abnormally increased, while in other studies MA was found at the upper limit of normal [59,62,64,65] or increased only in certain patients [55,63]. For reaction time (R), 5 studies found decreased values from reference ranges [57] or from healthy volunteers [52,53,55,58], 4 reported decreased kinetics reaction K parameter as compared with healthy volunteers [52,53,55,58], and 11 reported increased α angle as compared with reference ranges (42,45–48,52,53) or healthy volunteers [54,57,58,59,60,64,65]. Impaired fibrinolysis was found in eleven studies, with ‘fibrinolytic activity’ at 30 min after maximum amplitude (LY30) reduced as compared with reference ranges in healthy volunteers [52,53], or even undetectable [51,54,57,58,59,60,62,63,64,65].

TEG Functional Fibrinogen (CFF) was assessed in four studies [59,61,62,65], showing an increase in maximum clot amplitude with a median CFF-MA ranging from 41 to 56 mm for all patients as compared with manufacturer’s reference range (15 to 32 mm), and with a negative skewness coefficient of −0.37 [59].

Increased fibrinogen component of clot strength was considered as the hallmark of hypercoagulability, associated with at least one of the following: a shorter reaction time R [52,53,55,57,58], a shorter kinetic time K [52,53,55,58], and an increased α angle [52,53,54,55,56,57,58,59,60,64,65]. This pattern was often associated with an impaired [52,53] or absent fibrinolysis [51,54,57,58,59,60,62,63,64,65].

In summary, three points are worthy of attention. First, the hypercoagulable pattern defined as just above was observed in both ICU and non-ICU COVID-19 patients [65]. Second, it was observed despite UFH or LMWH anticoagulation at prophylactic doses or higher, and an anti-Xa activity within the target range without heparin neutralization (TEG, CK assay) [49]; and third it persisted over time [53,59,60,61]. However, the association between the observed pattern and the occurrence of thrombotic events remains a matter of debate. One study including 21 patients reported that an increase in MA provides 100% sensitivity and 100% negative predictive value to discriminate between patients with high or low rate of thrombotic events (44), but only a few patients were reported. Another study (44 patients) reported that no evidence of clot lysis at 30 min (LY30) associated with high D-dimers levels (>2600 µg/L) could predict thromboembolic events (*p* = 0.008) and need for hemodialysis in critically ill patients (*p* = 0.004) with COVID-19 [51]. However, seven other studies [53,55,58,59,60,63,64] comprising 243 patients did not find an association between the VET parameters and the occurrence of thrombotic events.

#### 3.5.3. Quantra

The Quantra device was used in two studies [66,67], both prospective, one of them comparing data from ICU non-COVID-19 patients with ICU COVID-19 patients [66]. The two studies included 44 ARDS intubated and mechanically ventilated ICU patients, of whom 33 were COVID-19 positive. All patients received anticoagulation according to local protocols or guidelines [74]. Tests were performed using the QPlus Cartridge, which contains heparinase in the CTH channel and polybrene in the CS and FCS channels to neutralize heparin. Results are summarized in Table 9.

Both studies suggested a hypercoagulable pattern associated with preserved thrombin generation, assessed by prothrombin fragments 1 + 2 and thrombin–antithrombin complex levels [66] and despite UFH or LMWH anticoagulation at a minimum of prophylactic dosing. However, the VET hypercoagulable pattern tended to normalize [67] after a 50% increase in thromboprophylaxis dosing and based on the body weight. However, the association between the documented hypercoagulable pattern and thrombotic event occurrence was not studied.

#### 3.5.4. ClotPro

ClotPro was used in a retrospective study in Austria [24] and in three cases in Hungary [68] of ICU patients who received anticoagulation at prophylactic doses or greater. VET assays were performed using four reagents and channels (Table A7), namely EX-test, IN-test, Fib-test, and tPA-test. Results are summarized in Table 10.

Results from the tPA-test showed a hypercoagulable pattern (increased maximum clot firmness) associated with impaired fibrinolysis; the latter was assessed either by a decreased lysis capacity of the clot in presence of tPA as compared with manufacturer’s reference values, followed few days later by a normalization with still marked elevated D-dimers levels [68], or by an increased clot lysis time as compared with healthy controls with a *p*-value < 0.01 [24]. However, these findings do not appear to be associated with the occurrence of thrombotic events [24].

## 4. Discussion

Although all of the studies share the common viscoelastometric testing concept in evaluating COVID 19 patients’ hemostasis, the differences in the testing systems and reagents, resultant data and implications, and variability of the patients’ severity of illness make interpretation difficult. The association with thrombotic events is not very well established, and might largely depend on the actual VET used. We will more specifically discuss whether VETs provide clinically relevant information about fibrinogen in a COVID-19 patient, and we will discuss its use regarding potential anticoagulation with heparins.

### 4.1. Methodological Issues in VET Studies

There are numerous methodological differences among the 44 studies using VETs to assess the hemostasis in COVID-19 patients we have retrieved and analyzed, explaining why results were not consistent through studies, or sometimes even conflicting. This was already raised by previously published reviews [70,71].

First, the design was heterogeneous among studies with 19 prospective studies [28,29,30,31,32,39,40,41,42,43,44,45,46,52,59,60,61,66,67], 19 retrospective ones [24,27,33,34,35,36,47,48,49,51,53,54,55,56,57,58,62,63,64], and 6 case reports [26,37,38,50,65,68] with no randomized controlled trial (VET versus no VET). Studies also differed on the timing of the sampling for VET assay (ranging from admission [27,29,30,31,36,37,38,44,45,46,50,53,56,66] to a median of 18 (13–29) days after admission [47]), the number of studied patients (ranging from 5 excepted case-reports [40,54] to 64 [58]), the anticoagulation regimen, and the diagnosis of thrombotic events (solely based upon clinical signs, based upon a systematic screening by imaging or based upon clinical signs and confirmed by imaging). There is variability how the authors defined hypercoagulable patterns in VETs based on the parameters used and the reference values considered. Some studies used reference range from local healthy subjects [24,28,31,32,33,39,40,41,46,47,50,52,53,55,56], while most of the reference values were manufacturer determined and could not be fully adapted to the local population and settings [1,3].

Second, there was also heterogeneity among the patients’ characteristics concerning age, severity of the disease, gender distribution, and comorbidities. In addition to the lack of power to evidence a statistically significant association between the VET patterns and thrombotic events, this heterogeneity could explain the differences between the studies’ results, at least in part.

One important consideration is different monitoring devices were used. Even though they share the same objective of viscoelastic clot properties evaluation, they present substantial differences from technological and methodological viewpoints. First, they rely on different technologies to monitor clot formation, clot strength, and clot lysis (i.e., thromboelastometry, thromboelastography, and sonorheometry). Second, there are some differences in the way the tests are carried out and the sample and reagents are delivered to perform the assay, specifically the activators. While in the most recent versions of the instruments (TEG6s, ROTEM sigma, Quantra, ClotPro) the reagents are already included in reaction cartridges or in tips and require only the addition of the blood sample, the previous versions (ROTEM gamma and delta, TEG5000) required manual or semi-automated pipetting of reagents and samples, resulting in very high inter- and intra-operator coefficients of variation for some parameters [77,78]. Third, the composition of the reagents differs from one manufacturer to another, especially for the assay aiming to assess the fibrinogen component of clot strength, also called functional fibrinogen. Briefly, clot strength is mainly due to the interaction between fibrin network (containing activated factor XIII), platelets [7], neutrophil extracellular traps [79], and red blood cells [80,81]. Platelets are an important contributor to the clot strength, and the MA (TEG), MCF (ROTEM and ClotPro), and CS (Quantra) parameters reflect both platelet count and function [9,82], as well as fibrin contribution. To assess functional fibrinogen, platelet contribution must be inhibited, and two different approaches are used that include abciximab (GpIIb-IIIa inhibitor, TEG and Quantra), cytochalasin D (cytoskeleton inhibitor, ROTEM), or a combination of both (ClotPro). Some studies [83,84,85] compared the fibrinogen contribution to clot mechanical strength measured with VET using either a GpIIb-IIIa inhibitor or cytochalasin D and showed that the latter was more efficient: functional fibrinogen assessment with TEG or Quantra could lead to an overestimation of fibrinogen levels as compared with ROTEM. However, cytochalasin D alone may not completely remove the platelet contribution, especially with a high platelet count, and a combination of a GpIIb-IIIa inhibitor and cytochalasin D seems to provide more accurate results [83,84,85].

Therefore, we can reasonably doubt that the results obtained from the different devices and studies are interchangeable, as previously noted in non-COVID-19 patients [3,86]. There may even be differences in results between different versions of the same device (as between ROTEM-delta and ROTEM-sigma for example [15,87], or between TEG5000 and TEGS6s [17,18,19]), but overall the devices show good correlations for the main parameters evaluated [14,86,88,89]. To our knowledge, there have not been previous comparisons in COVID-19 patients. Studies on VET have always been plagued by those issues, unfortunately still unresolved.

### 4.2. Definition of a Hypercoagulable State by VET and Association with Thrombotic Events

The conventional clotting time corresponds to the ‘reaction time’ R for TEG, and the ‘clotting time’ CT for ROTEM, ClotPro, and Quantra. Extended fibrin polymerization is described as the kinetics time K and α angle for TEG and CFT and α angle for ROTEM and ClotPro. The clot strength is defined as maximal mechanical strength (maximal amplitude MA for TEG, maximal clot firmness MCF for ROTEM and ClotPro, and clot stiffness CS for Quantra).

Beyond a purely biological definition, for which there is no consensus or appropriate term, sometimes ‘procoagulant’ or hypercoagulable, what matters is the association with the patient’s thrombotic risk. Outside the COVID-19 setting, two systematic reviews and a subsequent meta-analysis involving 1285 patients with solid tumors or hematopoietic malignancies [90] or 8944 surgical patients [91] showed that the occurrence of thrombotic events was associated with features consistent with hypercoagulability: acceleration of fibrin polymerization (increase in α angle in both ROTEM and TEG, shortened CFT in ROTEM and shortened K time in TEG) and increased clot mechanical strength (increase in MCF for ROTEM and in MA for TEG). However, another meta-analysis of 1081 patients in a variety of clinical settings [92] showed that ROTEM and TEG had a moderate ability to discriminate between patients who developed a thrombotic event and those who did not, with a diagnostic odds-ratio of 3.6, a low sensitivity (56%) but a somewhat better specificity (76%). It is noteworthy that the performance in the prediction of thrombotic events depends both on the type of device (with a better performance for ROTEM with a diagnostic odds-ratio of 6.3 against 3.2 for TEG), and on the type of thrombotic event (with a sensitivity of 67%, a specificity of 72%, and a diagnostic odds-ratio of 6.4 for arterial thrombotic events, contrasting with a sensitivity of 41%, a specificity of 70%, and an odds-ratio diagnosis of 3.1 for venous thrombotic events). Why VET findings should be more associated with arterial thrombotic events than with venous ones is obscure, though.

Regarding the 44 studies we examined, all authors concluded that COVID-19 patients displayed a hypercoagulable pattern characterized by an increased clot mechanical strength (assessed by CS in Quantra, MA in TEG and MCF in ROTEM and ClotPro) basically due to an excessive fibrin(ogen) component (assessed by FCS in Quantra, CFF-MA in TEG, FIBTEM-MCF in ROTEM and MCF from FIB-test in ClotPro), associated with a shortening of clot initiation (decreased K in TEG and CFT in ROTEM and ClotPro) in 18 studies [26,27,31,32,33,38,41,42,43,44,45,46,49,50,52,53,55,58], an acceleration of fibrin polymerization (increased α angle in TEG, ROTEM and ClotPro) in 15 studies [32,38,39,50,52,53,54,55,56,57,58,59,60,64,65], and an impaired or reduced fibrinolysis in 26 studies [24,26,30,32,34,37,39,40,42,43,44,46,48,51,52,53,54,57,58,59,60,62,63,64,65,68]. It is crucial to note however that an association between that pattern and thrombotic events was evidenced by only one study [56] out of the sixteen addressing the issue [24,31,33,34,35,44,49,51,53,55,56,58,59,60,63,64]: an increase in the maximum clot amplitude (MA) provides 100% sensitivity and 100% negative predictive value to discriminate between patients with a high or low rate of thrombotic events, but confidence intervals were not reported [56]. This raises doubts about the clinical significance of the ‘so called’ hypercoagulability identified by VET and its potential clinical implications (e.g., thrombotic risk stratification or adjustment of thromboprophylaxis).

### 4.3. Ability of VETs to Detect Hypofibrinolysis State and Association with Thrombotic Events

Fibrinolysis is monitored at a specific time x minutes after MA was reached for TEG (LY(x) parameter), and by maximal lysis ML (reduction in clot firmness after MCF in relation to MCF) or lysis of the clot at a given time x minutes after CT was reached (LI(x)) parameter for ROTEM and ClotPro. The diminution in clot maximum amplitude was thought to be due to both fibrinolysis and potentially platelet-mediated clot retraction [4,5,6,93]. However, as no change in clot mechanical strength after the maximum was reached was reported in many studies in COVID-19 patients, platelet-mediated clot retraction does not seem to play a significant role here.

Usually VETs are used to detect major hyperfibrinolytic states [94] that occur in the most severe, advanced stages of hemostasis derangements in clinical settings such as trauma and perioperative hemorrhage. However, could they be used to assess hypofibrinolysis? VETs have shown potential usefulness in sepsis-induced coagulopathy [10] and trauma-induced coagulopathy [11] to detect low levels of fibrinolysis and to identify patients for whom the administration of tranexamic acid should be avoided. Endogenous systemic fibrinolysis is usually weak because of low or even no circulating levels of free plasminogen activators, which are fully complexed to PAI-1 and thus inactive. Normal lysis of a whole blood clot is therefore a slow phenomenon [95], and its visualization on a VET trace recorded during one hour or two seems unlikely. Furthermore, as the zero value belongs to the manufacturer’s reference range, speaking about a reduced or an absent fibrinolysis seems awkward if there is no control group for comparison. Among the 25 studies reporting a reduced or absent fibrinolysis, only 8 [24,32,39,40,44,48,52,53] made this assessment by comparison with a control group.

Several ROTEM and TEG modifications have been reported adding urokinase plasminogen activator (uPA) or tissue plasminogen activator (tPA) to demonstrate hypofibrinolysis. A brief literature search revealed multiple protocols for modified ROTEM and TEG including addition of a plasminogen activator. Although they show evidence of hypofibrinolysis in different clinical settings, they all share the same methodological issues and limitations. First, there is a lack of standardization concerning tissue factor concentrations, as low levels added to the sample produce non-reproducible results and often a weak clot [96,97]. Second, there is also a lack of standardization in tPA concentrations studied ranging from 50 to 625 ng/mL [96,97,98], and similar results for modified VET with uPA [99,100], with an ‘optimal concentration of uPA’ differing from subject to subject, and a wide interindividual variation in lysis parameters [99].

Among the 44 studies analyzed in this review, only four investigated the effect of adding tPA to standard VET. Two used the ClotPro device [24,68] and its ready-to-use tPA-test reagents, which are now CE-marked, whereas the two others [39,40] used an in-house ROTEM assay with two different tPA concentrations (named TEM-tPA), making a comparison between them problematic. No study has investigated a defective fibrinolysis using the Quantra device into the COVID-19 context, whereas a new dedicated reagent cartridge is now available [23]. The four articles share the same conclusion that increased clot maximum amplitude and decreased lysis index reflect an increase in clot strength and a decreased fibrinolytic capacity, results that need to be confirmed with a larger cohort. Further, the TEM-tPA assay needs to be standardized and validated [101], although it seemed to show good intra- and inter-assay precision in healthy controls [39].

Association between impaired fibrinolysis assessed with VETs and clinical outcomes is a matter of debate. Some studies failed to find an association [24,53,55,58,59], while others suggested that impaired fibrinolysis was associated with a higher rate [34] and a shorter time to the occurrence of thrombotic events [51], and together with D-dimer levels it could predict thrombotic events [44,51] and the need for hemodialysis in critically ill patients with COVID-19 [51].

### 4.4. Correlation between Clauss Fibrinogen and Functional Fibrinogen Assessed by VETs

Outside of the COVID-19 context, the ROTEM FIBTEM is the most studied point of care fibrinogen level assay with numerous studies in trauma, cardiac surgery, liver transplantation, and obstetrics. The correlation between Clauss fibrinogen and FIBTEM-MCF or fibrinogen-related TEG parameters was reported as variable with R^2^ values ranging from 0.44 to 0.94 for ROTEM [102] and from 0 [103] to at least 0.80 [102] for TEG. The clinical experience with the Quantra device is limited, with only few published studies to date, but the correlation between Clauss fibrinogen and FCS ranged from moderate to very good, with R^2^ values ranging from 0.55 to 0.88, with a huge variability between studies [14,22,104,105]. To our knowledge, data concerning correlation between Clauss fibrinogen and clot amplitude and maximum clot firmness provided by the ClotPro FIB-test assay is not yet available.

Among the 44 studies dealing with VETs and COVID-19 patients, only a few ones [33,41,53,58] investigated the correlation between Clauss fibrinogen and ‘functional fibrinogen’ assessed by VETs. For ROTEM® (FIBTEM), one report noted a good correlation (Pearson’s correlation coefficient r = 0.84) [41], while another showed no correlation (*p* = 0.130) in children [33]. Two studies explored TEG in this regard and reported a moderate to good association (Pearson’s correlation coefficient r = 0.453 [58] and 0.74 [53]). These limited results due to low COVID-19 patient numbers and different assays suggest more studies are required.

Further, whether VET characteristics are unique to hyperfibrinogenemia alone is an important question, as almost all COVID-19 patients also present with hyperfibrinogenemia. Patients with hyperfibrinogenemia may exhibit a ‘functional fibrinogen’ (VET parameter) in the reference range [35,37,66], while other reports of fibrinogen levels within reference ranges exhibit increased functional fibrinogen [26,28,48].

The authors of a previously published review [69] highlighted the potential usefulness of VET in accurately assessing plasma fibrinogen levels in COVID-19 patients receiving direct thrombin inhibitors (DTI) through the assessment of the clot amplitude of the functional fibrinogen assay. Indeed, evaluation of fibrinogen levels by the Clauss method could lead to an underestimation due to the inhibition of the thrombin included in the reagent by the DTI [106,107], ranging from 23 to 96% according to the reagent used [107].

### 4.5. Impact of Differences in Anticoagulation Regimens (Type (UFH, LMWH) and Dosage)

Most currently studied COVID-19 patients receive heparin (LMWH or UFH), either with prophylactic or therapeutic regimens according to local protocol or guidelines [74,75,76,108]. Some studies specifically noted when blood samples for VETs were drawn in heparinized patients, but the timing of administration was often missing as well as anti-Xa levels. While for TEG, heparinase reagents were frequently used (14 studies of 15 [51,52,53,54,55,56,57,58,59,60,62,63,64,65]), this was not the case for ROTEM: only a few ones (4 of 25) generated data with HEPTEM assay, together with INTEM assay. Among these four latter studies, only one [26] reported different results from the two assays, whereas the three others showed similar results with both assays [41,44,50]. This raises questions that include (i) the effect of heparin, particularly at low doses, on VET results, (ii) whether heparinase or polybrene added to heparinized blood completely neutralized circulating heparin, and (iii) whether VETs can be used to guide heparin therapy. These questions were not raised by the previously published reviews [69,70,71,72].

The effect of heparin (UFH and LMWH) on VETs (performed without heparin neutralization) seems, according to the literature, to depend mainly on the heparin dose and the VETs used. Two trends have emerged for prophylactic or therapeutic concentrations (anti-Xa up to 1.5 IU/mL). First, with TEG, anti-Xa levels and R and K parameters (clot initiation) seemed to correlate, while an inverse correlation between anti-Xa levels and α angle and MA is observed (fibrin polymerization), sometimes leading to a ‘flat line’ with the highest anti-Xa levels [109,110,111,112]. Second, fewer data are available for ROTEM, but there seems to be a correlation only between anti-Xa levels and CT parameter from the INTEM assay (clot initiation) [112,113,114].

In the setting of cardiac surgery with cardiopulmonary bypass, VETs have been increasingly used, and manufacturers have adapted testing to neutralize circulating heparin by the addition of heparinase or polybrene in order to differentiate between insufficient heparin neutralization in patients with protamine from underlying post-bypass coagulopathy. Few data are available, however, on whether this neutralization is complete. In an in vitro study performed with TEG and coated cups with heparinase [109], results were similar between native samples and samples spiked with heparin (UFH or LMWH) or danaparoid, but tested concentrations were too low (0.005 to 0.05 IU/mL) to be clinically relevant. Another in vitro study performed with ROTEM and heparinase [113] showed similar results between native samples and samples spiked with increasing heparin (HNF) concentrations ranging from 0.1 to 1 IU/mL.

Among the 44 COVID studies we retrieved, several mentioned a considerably high incidence of thrombotic events despite thromboprophylaxis, in line with most reports, and raising the potential interest of increasing anticoagulant doses. Could VETs then be useful to identify patients who will benefit from a higher dose of thrombosis anticoagulant prophylaxis? This remains a matter of debate. According to the authors, VETs seem able to detect coagulation abnormalities advocating for a hypercoagulable prothrombotic state in a broad sense, including procoagulable state (with a decreased clot formation time), hypercoagulable state (with an increased clot strength), and impaired fibrinolysis (with a reduced or absent clot lysis) early in the course of the disease, and even if conventional coagulation tests remain in the reference ranges. Second, as reduced or absent fibrinolysis was associated with an increased risk of thrombotic events outcome despite anticoagulation [34,42,43,44,51,54,57,63,64], we could assume that VET results might be used to adapt level of anticoagulation. Five studies [26,47,59,61,67] have reported VET parameters from ICU COVID-19 patients before and after an intensification of thromboprophylaxis, but results were inconsistent. Two of them showed a decrease in clot mechanical strength and in functional fibrinogen level [47,67], and even in the rate of thrombotic events [47], and the three others [26,59,61] did not find any significant difference. However, if VETs still demonstrate a hypercoagulable pattern despite anticoagulation at least with a prophylactic dose and even an anti-Xa level within the target range [60,61], it was not always associated with thrombotic outcomes, although there was no systematic VTE screening either [28,35,58,63]. Third, three studies have shown an exaggerated thrombin generation despite anticoagulation, at least with a prophylactic dose [39,48,66], advocating for a new way to monitor efficiency of thromboprophylaxis.

Rather than VETs, the study of thrombin generation could be more interesting to adjust anticoagulant therapy as heparin inhibits thrombin generation by multiple pathways as reviewed elsewhere [115]. Several methods exist to study thrombin generation, either with biomarkers such as prothrombin fragment 1 + 2 or thrombin–antithrombin complexes [116] (thrombin generation in vivo), or in vitro by assessment of the levels of thrombin over time (through the use of a chromogenic or fluorogenic substrate) in response to initiation of coagulation. Regarding the latter, several commercial devices and assays are available [117,118]. Of note, thrombin generation assays (TGAs) are highly sensitive to preanalytical aspects [119]. So far, some in vitro studies have reported that there was a heparin concentration dependent decrease in thrombin generation [120,121]. Studies showed that COVID-19 patients had a higher endogenous thrombin potential [31,39,48,66,122,123,124,125,126,127] than manufacturer’s reference range, healthy controls or patients with sepsis, sometimes despite UFH or LMWH anticoagulation at a minimum of prophylactic dosing. Few studies found a heparin dose-dependent decrease in thrombin generation [125,127] as described in vitro. Interestingly, one study showed that a persisting thrombin burst despite anticoagulation correlated with non-survival [123], whereas another found no difference between noncritical and critically ill COVID-19 patients [124]. Further studies are needed to evaluate the clinical value in this context of the in vitro study of thrombin generation, and particularly with the new automated ST Genesia device [118], as already mentioned elsewhere [128]. A potential issue could be the non-availability of such device and the high turn-around-time for a result There is also an unmet need regarding the exploration of fibrin polymerization and lysis [95] with good and convenient assays.

### 4.6. Summary of the Conclusions of the Previously Published Reviews 

Conclusions of the previously published reviews are summarized in Table 11. Overall, the four reviews reported the same findings as we do: COVID-19 patients displayed an abnormal VET pattern [69,70,71,72], but further studies are needed for various reasons. Moreover, we challenge the idea that such a pattern represents hypercoagulability; one main reason is that inhibitory systems are not at all taken into account, in sharp contrast with TGAs.

Of note, except the systematic review about the potential usefulness of TEG [71], no consistent association between the abnormal VET pattern and clinical outcome could have been demonstrated. Interestingly, one review [69] pointed out the potential usefulness of VETs in accurately assessing plasma fibrinogen levels in COVID-19 patients receiving DTI (see Section 4.4).

## 5. Conclusions

VETs are now well established in acute settings to assist in bleeding management and transfusion practices, with convenient, fully automated devices and ready-to-use reagents. Since the beginning of the pandemic, they were used to characterize hemostasis abnormalities in critically ill COVID-19 patients. As already reported in previous reviews [69,70,71,72], almost all the studies we analyzed reported increased clot strength, considered to be a hallmark of the ‘hypercoagulable state’, often associated with impaired fibrinolysis (with the analytical limitations we have emphasized)—globally referred to as ‘prothrombotic pattern’, but there was no consistent association with clinical outcomes. Indeed, few studies suggested an association with the occurrence of thrombotic events, as well as with the need for hemodialysis [34,44,51,56]. However, lack of power (low number of studied patients), retrospective design, and no standardized study protocol are of concern.

In the COVID-19 setting, the appraisal of (high) fibrinogen levels through VET as opposed to the Clauss method in the laboratory is not an obvious asset. As already pointed out however [69], VET could be of interest for accurately assessing plasma fibrinogen levels in COVID-19 patients receiving DTI through the assessment of the clot amplitude in functional fibrinogen assay. Modified VETs (with addition of a plasminogen activator) to detect, quantify, and monitor hypofibrinolysis in whole blood (with the advantage for instance to integrate the PAI-1 released by platelets) could be of clinical relevance [24,39,40,68].

Three different types of studies would be needed. First, prospective ones comparing the results from the different available devices are needed. Second, as it was already highlighted by previously published reviews [69,70,71,72], further prospective studies are needed, ideally randomized, to highlight the added-value of VET in predicting the clinical course of the disease, addressing patients to the appropriate ward according to their risk stratification, and identifying which patients would benefit from intensified anticoagulant treatment and those who would show clot resistance to fibrinolysis. Third, prospective randomized controlled trials are needed to evaluate the usefulness of VET and TGA in monitoring and adapting thromboprophylaxis.

## Figures and Tables

**Table 1:** Eligibility criteria.

PICOS	Inclusion	Exclusion
Participants	All patients with confirmed COVID-19 infection regardless of age	Pregnancy Pre-existing coagulation disorder
Intervention	Viscoelastometric testing performed	-
Comparison	Reference values (manufacturer’s based or healthy controls) ICU COVID-19 patients and non-ICU COVID-19 patients ICU COVID-19 patients and ICU non-COVID-19 patients	-
Outcomes	VET parameters in COVID-19 patients Difference in VET parameters between ICU COVID-19 patients and non-ICU COVID-19 patients Difference in VET parameters between ICU COVID-19 patients and ICU non-COVID-19 patients Association between VET parameters and clinical outcomes Association between VET parameters and Clauss fibrinogen	-
Study design	Randomized controlled trials Observational clinical studies Case reports	Opinion papers Review papers Healthcare guidelines Protocol Non-human or in vitro studies

Abbreviations: VET: viscoelastometric testing; ICU: Intensive care unit.

**Table 2:** Characteristics of the reviews already published.

First Author (Title)	Type of the Review	Aim of the Review	Number and Type of Studies Included	VET Devices
Görlinger et al. [69] (COVID-19 associated coagulopathy and inflammatory response: what do we know already and what are the knowledge gaps?)	Narrative review	Review of coagulation abnormalities and inflammatory response associated with COVID-19	8 studies (5 prospective, 3 retrospective)	ROTEM, TEG, Quantra
Tsantes et al. [70] (COVID-19 Infection-Related Coagulopathy and Viscoelastic Methods: A Paradigm for Their Clinical Utility in Critical Illness)	Narrative review	Evaluation of the usefulness of VETs in clinical practice to guide anticoagulant treatments or predict prognosis	13 studies (8 prospective, 5 retrospective)	ROTEM, TEG, Quantra
Hartmann et al. [71] (The Role of TEG Analysis in Patients with COVID-19-Associated Coagulopathy: A Systematic Review)	Systematic review	Evaluation of the usefulness of TEG in clinical practice to identify and manage hypercoagulation associated with COVID-19	15 studies (5 prospective, 9 retrospective and one case report)	TEG
Słomka et al. [72] (Hemostasis in Coronavirus Disease 2019-Lesson from Viscoelastic Methods: A Systematic Review)	Systematic review	Evaluation of the performance of TEG and TEM in the assessment of blood coagulation and fibrinolysis in patients with COVID-19	10 studies (2 prospective, 8 retrospective)	ROTEM, TEG

**Table 3:** Characteristics of the included studies.

First Author (Country)	Device	Study Design	Ward	*n*	Number of Patients with Viscoelastic Test Performed	Timing of Assay	Number of Patients with Invasive Mechanical Ventilation (*n*)	Number of Patients under ECMO (*n*)	Number of Patients with Renal Replacement Therapy (*n*)	Age ^1^	Number of COVID-19 Patients with Thrombotic Events	Diagnosis of Thrombotic Events	Anticoagulation
Iwasaki et al. (Japan) [26]	ROTEM (NS)	Case report	ICU	1	1	1 day after ICU admission	1	NP	NP	57	None	NP	None until TE, then UFH 10,000 IU/d
Pavoni et al. (Italy) [27]	ROTEM gamma	Retrospective observational study	ICU	40	40	ICU admission, then 5 and 10 days later	4/40	NP	NP	61 ± 13	20/40 patients (6 DVT, 2 TE, 12 catheter related thrombosis)	Systematic screening from common femoral vein by ultrasound	Enoxaparin 40–60 mg/d according to local protocol
Boscolo et al. (Italy) [28]	ROTEM delta	Prospective observational study	ICU	32	32	NP	21/32	NP	NP	68 (62–75)	11/32 patients	No systematic screening	NP
			IMW	32	32		None	None	None	61 (53–71)	3/32 patients		
Corrêa et al. (Brazil) [29]	ROTEM delta	Prospective observational study	ICU	30	30	ICU admission, then 1, 3, 7 and 14 days later	27/30	NP	10/30	61 (52–83)	6/30 patients (4 DVT, 2 PE)	NP	At least prophylactic UFH or LMWH
Madathil et al. (USA) [30]	ROTEM delta	Prospective observational study	ICU	11	11	ICU admission, then 24–48 h later	11/11	NP	NP	53 (45.5–65.5)	NP	NP	NP
Spiezia et al. (Italy) [31]	ROTEM delta	Prospective observational case control study	ICU	22	22	ICU admission	19/22	NP	NP	67 ± 8	5/22 patients (DVT)	NP	Prophylactic LMWH
Tsantes et al. (Greece) [32]	ROTEM delta	Prospective observational study	ICU COVID-19 patients	11	11	NP	NP	NP	NP	78 (67–71)	NP	NP	Enoxaparin 1 mg/kg bid
			ICU non COVID-19 patients	9	9		NP	NP	NP	NP			Enoxaparin 1 mg/kg od
			IMW COVID-19 patients	21	21		NP	NP	NP	73 (50–88)			Enoxaparin 1 mg/kg od
Al-Ghafry et al. (USA) [33]	ROTEM delta	Retrospective observational study	PICU (*n* = 5) and PW (*n* = 3)	8	8	1 to 4 days after hospital admission	None	None	None	12.9 (2–20)	None	NP	Prophylactic enoxaparin 0.5 mg/kg bid according to oxygen requirement and D-dimers levels, escalated to therapeutic dose (1 mg/kg bid) if clinical deterioration
Creel-Bulos et al. (USA) [34]	ROTEM delta	Retrospective observational study	ICU	25	25	NP	NP	NP	NP	63 (53–77)	9/25 patients (7 DVT, 4 PE, 1 arterial thrombosis)	Ultrasound or CT imaging based on clinical suspicion	Prophylactic LMWH or UFH
Hoechter et al. (Germany) [35]	ROTEM delta	Retrospective observational case control study	ICU COVID-19 pneumonia	22	11	Within 48 h after ICU admission	22/22	NP	NP	64 (52–70)	NP	NP	Prophylactic UFH according to local guidelines
			ICU non COVID-19 pneumonia	14	14	NP	14/14	NP	NP	49 (36–57)			
Roh et al. (USA) [36]	ROTEM delta	Retrospective observational case control study	ICU	30	30	ICU admission	NP	NP	NP	63 ± 12	10/30 patients (3 DVT, 1 PE, 1 both DVT and PE, 4 arterial thrombosis, 1 both arterial thrombosis and DVT)	Ultrasound or CT imaging based on clinical suspicion	At least prophylactic UFH or LMWH
Kong et al. (United Kingdom) [37]	ROTEM delta	Case report	ICU	1	1	2 h after ICU admission	No	No	No	48	None	NP	None until ROTEM analysis
			ICU	1	1	NP	1	No	1	68	None		NP
Raval et al. (USA) [38]	ROTEM delta	Case report	ICU	1	1	ICU admission	1	No	No	63	None	NP	None at admission, then UFH 7500 IU/8 h
Nougier et al. (France) [39]	Modified ROTEM delta (TEM-tPA)	Prospective observational case control study	ICU	40	19	NP	33/40	NP	7/40	62.8 ± 13.1	14/40 patients (8 PE, 5 DVT, 1 arterial thrombosis)	Ultrasound or CT imaging based on clinical suspicion	At least prophylactic UFH or LMWH
			IMW	38	4		None	None	None	60.2 ± 14.6	NP		
Weiss et al. (France) [40]	Modified ROTEM delta (TEM-tPA)	Prospective observational case control study	ICU	5	5	NP	NP	NP	NP	57 ± 15	3/5 patients	NP	Thromboprophylaxis according to current guidelines
Almskog et al. (Sweden) [41]	ROTEM sigma	Prospective observational study	ICU	20	20	1 day after hospital admission	NP	NP	NP	62 (55–66)	NP	NP	At least prophylactic tinzaparin
			IMW	40	40		NP	NP	NP	61 (51–74)			
Collett et al. (Australia) [42]	ROTEM sigma	Prospective observational study	ICU	6	6	NP	5/6	None	2/6	69 (64.2–73)	3/6 patients (1 PE, 1 catheter related thrombosis, 1 TE not clinically suspected)	NP	Enoxaparin 40 mg od
Ibañez et al. (Spain) [43]	ROTEM sigma	Prospective observational study	ICU	19	19	24–48 h after ICU admission	NP	NP	NP	61 (55–73)	5/19 patients (2 DVT, 2 PE, 1 arterial thrombosis)	NP	Enoxaparin 40–80 mg/d according to local protocol
Kruse et al. (Germany) [44]	ROTEM sigma	Prospective observational study	ICU	40	40	ICU admission	31/40	10/40	21/40	67 (57.3–76.6)	23/40 patients (14 DVT, 4 PE, 3 ischemic stroke, 1 clotted ECMO cannula, 1 complete thrombosis of the ECMO circuit)	Systematic screening by ultrasound once a week	At least prophylactic LMWH (or argatroban if ECMO)
Pavoni et al. (Italy) [45]	ROTEM sigma	Prospective case controls observational study	ICU COVID-19 pneumonia	20	20	ICU admission, then 5 and 10 days later	2/20	NP	NP	60.3 ± 15.2	NP	NP	Enoxaparin 40–60 mg/d according to local protocol
			ICU non COVID-19 pneumonia	25	25		8/25	NP	NP	66.5 ± 18.8	NP		
Spiezia et al. (Italy) [46]	ROTEM sigma	Prospective case controls observational study	IMW COVID-19 pneumonia	56	56	Within 6 h after hospital admission	NP	NP	NP	64 ± 15	NP	NP	NP
			IMW non COVID-19 pneumonia	56	56					76 ± 11	NP		
Van der Linden et al. (Sweden) [47]	ROTEM sigma	Cross-sectional study	ICU before enhanced anticoagulation	12	12	13 (7–16) days after ICU admission	12/12	NP	6/12	54 ± 9	7/12 patients (6 PE, 1 DVT)	Ultrasound or CT imaging based on clinical suspicion	LMWH 129 ± 53 IU/kg/24 h or UFH infusion
			ICU after enhanced anticoagulation	14	14	18 (13–29) days after ICU admission	14/14	NP	8/14	59 ± 8	5/14 patients (3 PE, 2 DVT)		LMWH 200 ± 82 IU/kg/24 h or UFH infusion
Blasi et al. (Spain) [48]	ROTEM sigma	Retrospective observational study	ICU	12	12	4 days after hospital admission	12/12	NP	NP	69 (57–76)	NP	NP	At least prophylactic LMWH
			IMW	11	11		None	NP	NP	58 (42–74)			
Van Veenendaal et al. (The Netherlands) [49]	ROTEM sigma	Retrospective observational study	ICU	47	47	NP	47/47	NP	NP	63 (29–79)	10/47 patients (10 PE)	Ultrasound or CT imaging based on clinical suspicion	At least prophylactic UFH or LMWH
Lazar et al. (USA) [50]	ROTEM sigma	Case report	IMW	1	1	Hospital admission	No	No	No	NP	NP	NP	None at admission, then prophylactic UFH
			IMW	1	1		No	No	No	NP	NP		None at admission, then enoxaparin 60 mg od
Wright et al. (USA) [51]	TEG (NS)	Retrospective observational study	ICU	44	44	NP	43/44	20/44	NP	54 (42–59)	11/39 TE, 6/39 thrombotic stroke, 16/39 acute renal failure requiring dialysis	Ultrasound or CT imaging based on clinical suspicion	At least enoxaparin 40–60 mg od or UFH 10,000–15,000 IU per day
Panigada et al. (Italy) [52]	TEG5000	Prospective observational study	ICU	24	24	NP	24/24	NP	NP	56 (23–71)	NP	NP	At least prophylactic dose of LMWH or UFH
Cordier et al. (France) [53]	TEG5000	Retrospective observational study	ICU	24	24	ICU admission, then at discharge from the ICU	NP	NP	NP	69 (61–71)	6/24 patients (4 isolated PE, 1 ischemic stroke, 1 both PE and ischemic stroke)	Ultrasound or CT imaging based on clinical suspicion	Thromboprophylaxis according to current guidelines
Hightower et al. (USA) [54]	TEG5000	Retrospective observational study	ICU	5	5	NP	4/5	None	None	59 (38–69.5)	2/5 patients	Ultrasound or CT imaging based on clinical degradation	Enoxaparin 40 mg od or therapeutic UFH
Maatman et al. (USA) [55]	TEG5000	Retrospective multi-center observational study	ICU	109	12	3.5 days after hospital admission	102/109	NP	16/109	61 ± 16	31/109 patients: 2/31 upon admission and 29/31 despite anticoagulation (26 isolated DVT, 1 isolated PE, 4 both DVT and PE)	Ultrasound or CT imaging based on clinical suspicion	UFH 5000 IU/8 h, 40 mg enoxaparin od or 30 mg enoxaparin bid
Mortus et al. (USA) [56]	TEG5000	Retrospective cohort study	ICU	21	21	ICU admission	NP	2/21	18/21	68 ± 11	13/21 patients for a total of 46 recorded events	NP	Standard DVT chemoprophylaxis upon admission with subsequent therapeutic anticoagulation (UFH or enoxaparin 2 mg/kg/d) if thrombotic complications
Sadd et al. (USA) [57]	TEG5000	Retrospective observational cohort study	ICU	10	10	2.5 days after ICU admission	10/10	NP	3/10	58 (49–70)	4/10 patients (3 AKI, 1 CRRT)	NP	Standard UFH or LMWH prophylaxis with subsequent therapeutic anticoagulation according to local guidelines
Yuriditsky et al. (USA) [58]	TEG5000	Retrospective observational study	ICU	64	64	Within 72 h after ICU admission	NP	NP	NP	64 (57–71)	20/64 TE, 31/64 acute renal failure	Ultrasound or CT imaging based on clinical suspicion	Standard UFH or LMWH prophylaxis with subsequent therapeutic anticoagulation according to D-dimers levels or if thrombotic events
Bocci et al. (Italy) [59]	TEG6s	Prospective observational study	ICU	40	40	Within 24 h after ICU admission, then 7 days later	29/40	NP	NP	67.5 (55–77)	2/40 patients (2 PE)	Ultrasound and CT imaging not routinely used	Full-dose anticoagulation according to local protocols (enoxaparin 0,5 mg/kg/12 h, UFH 7500 IU/8 h or UFH infusion)
Stattin et al. (Sweden) [60]	TEG6s	Prospective observational study	ICU	31	31	NP	24/31	NP	NP	65 (51–70)	5/31 patients	NP	Prophylactic dalteparin (75–100 IU/kg) with anti-Xa levels target 0.2–0.4 IU/mL
Vlot et al. (The Netherlands) [61]	TEG6s	Prospective observational study	ICU	16	16	NP	16/16	NP	6/16	67 (56–73)	None	No systematic screening	Increase prophylactic dose of LMWH: nadroparin 5700 IU bid (or 7600 IU according to body weight) instead of 2850 IU od
Patel et al. (United Kingdom) [62]	TEG6s	Retrospective observational study	ICU	39	39	NP	39/39	20/39	NP	52.5 (29–79)	15/39 patients with acute PE, 4/22 with DVT	Systematic screening by CT pulmonary angiography	At least prophylactic dose of LMWH or UFH with anti-Xa levels of 0.2–0.3 IU/mL
Salem et al. (United Arab Emirates) [63]	TEG6s	Retrospective observational study	ICU	52	52	NP	46/52	7/52	16/52	53 (39–62)	14/52 patients (8 DVT, 6 PE, 2 arterial thrombosis)	NP	Standard UFH or LMWH prophylaxis with subsequent therapeutic anticoagulation according to local guidelines
Shah et al. (United Kingdom) [64]	TEG6s	Multicenter retrospective observational study	ICU	187	20	NP	166/187	6/187	80/187	57 (49–64)	81/187 patients (42 PE, 22 DVT, 25 arterial thrombosis)Extracorporeal circuit disruption *n* = 23	Ultrasound or CT imaging based on clinical suspicion	Standard weight-based LWMH prophylaxis with subsequent therapeutic anticoagulation if thrombotic events
Fan et al. (Singapore) [65]	TEG6s	Case report	IMW	1	1	13 days after admission, 1 h after clinical sign of TE	No	No	No	39	1	Ultrasound or CT imaging based on clinical suspicion	None until TE, then therapeutic UFH 1300 IU/h (anti-Xa levels 0.4–0.6 IU/mL)
Masi et al. (France) [66]	Quantra	Prospective single-center cohort study	ICU COVID-19 ARDS	17	17	ICU admission	17/17	NP	NP	48 (42–58)	3/17 patients (3 PE)	NP	Thromboprophylaxis according to current guidelines
			ICU non COVID-19 ARDS	11	11		11/11	NP	NP	34 (28–55)	NP		NP
Ranucci et al. (Italy) [67]	Quantra	Prospective observational study	ICU	16	16	2–5 days after ICU admission, then 14 days after	16/16	NP	NP	61 (55–65)	None	NP	Nadroparin 4000 IU bid then 6000 or 8000 IU bid according to BMI
Bachler et al. (Austria) [24]	ClotPro	Retrospective study	ICU	20	20	8.5 (4.5–15) days after ICU admission	NP	NP	NP	61.5 (56.25–68)	2/20 patients	NP	Enoxaparin 80 (60–100) mg/day (*n* = 16) or argatroban (*n* = 4)
Zátroch et al. (Hungary) [68]	ClotPro	Case report	ICU	1	1	NP	No	No	No	62	1	NP	Enoxaparin 80 mg bid
				1	1	NP	1	No	1	80	1		Enoxaparin 60 mg od
				1	1	NP	1	No	No	84	1		Enoxaparin 20 mg od

^1^ Variables are reported as number, as median with interquartile range (median (IQR)) or as mean with standard deviation (mean ± SD). Abbreviations: ICU: Intensive care unit; IMW: Internal medicine ward; PICU: Pediatric intensive care unit; PW: Pediatric ward; UFH: Unfractionated heparin; LMWH: Low molecular weight heparin; od: once a day; bid: twice a day; IMV: Invasive mechanical ventilation; ECMO: Extracorporeal membrane oxygenation; RRT: Renal replacement therapy; CRRT: Continuous renal replacement therapy; TE: Thrombotic events; DVT: Deep vein thrombosis; PE: Pulmonary embolism; AKI: Acute kidney injury; NP: Not provided; NS: Not specified; TEG: Thromboelastography; ROTEM: Rotational thromboelastometry; TEM: Thromboelastometry; tPA: tissue plasminogen activator.

**Table 4:** Characteristics of the included patients.

First Author (Country)	Device	*n*	Ward	Age	M:F Ratio	SOFA Score	APACHE II Score	SAPS II Score	SAPS III Score	DIC Score	SIC Score	BMI (*18.5–24.9* kg/m^2^)	Comorbidities	CRP (mg/L) (*<5* mg/L) *	Fibrinogen (mg/dL) (200–400 mg/dL) *	D-Dimers (µg/L)	Platelets (10^3^/µL) (*150–450 × 10^3^*/µL) *
Iwasaki et al. (Japan) [26]	ROTEM (NS)	1	ICU	57	F	NP	NP	NP	NP	NP	NP	NP	NP	391	334	1500	203
Pavoni et al. (Italy) [27]	ROTEM gamma	40	ICU	61 ± 13	24 M: 16 F	4 ± 1	NP	NP	NP	NP	NP	28.4 ± 4.7	Yes ^5^	NP	896 ± 110	1556 ± 1090	318 ± 168
Boscolo et al. (Italy) [28]	ROTEM delta	32	ICU	68 (62–75)	26 M: 6 F	3 (3–6)	NP	NP	NP	1 (0–2)	2 (2–2)	29 (27–32)	NP	110 (55–167)	500 (450–570)	315 (164–1326)	283 (194–336)
		32	IMW	61 (53–71)	24 M: 8 F	2 (1–2)	NP	NP	NP	0 (0–1.8)	2 (1–2)	29 (24–32)		46 (16–96)	450 (330–530)	263 (193–598)	234 (197–290)
Corrêa et al. (Brazil) [29]	ROTEM delta	30	ICU	61 (52–83)	15 M: 15 F	10 (7–12)	NP	NP	49 (41–61)	/	/	29.3 (24.4–32.2)	Yes ^10^	NP	600 (480–680)	1287 (798–2202)	226 (176–261)
Madathil et al. (USA) [30]	ROTEM delta	11	ICU	53 (45.5–65.5)	7 M: 4 F	NP	NP	NP	NP	NP	NP	28.1 (27.1–34.6)	Yes ^11^	NP	NP	NP	NP
Spiezia et al. (Italy) [31]	ROTEM delta	22	ICU	67 ± 8	20 M: 2 F	4 ± 2	NP	NP	NP	NP	NP	30 ± 6	Yes ^4^	NP	517 ± 148	5343 ± 2099	240 ± 119
Tsantes et al. (Greece) [32]	ROTEM delta	11	ICU COVID patients	78 (67–71)	10 M: 1 F	NP	NP	NP	NP	NP	NP	NP	NP	48 (23–128)	439 (313–440)	2420 (1470–7320)	262 (120–350)
		9	ICU non COVID patients	NP	NP	NP	NP	NP	NP	NP	NP	NP		NP	NP	NP	NP
		21	IMW COVID patients	73 (50–88)	11 M: 10 F	NP	NP	NP	NP	NP	NP	NP		32 (9–55)	437 (399–503)	860 (540–1210)	253 (207–396)
Al-Ghafry et al. (USA) [33]	ROTEM delta	8	PICU (*n* = 5) and PW (*n* = 3)	12.9 (2–20)	4 M: 4 F	NP	NP	NP	NP	NP	NP	21.9 (13.3–31.9)	NP	86 (4–130)	540 (329–732)	932 (151–2451)	258 (104–446)
Creel-Bulos et al. (USA) [34]	ROTEM delta	25	ICU	63 (53–77)	NP	NP	NP	NP	NP	NP	NP	NP	NP	276 (229–326)	NP	7287 (4939–23,912)	NP
Hoechter et al. (Germany) [35]	ROTEM delta	22	ICU COVID+ (ROTEM *n* = 11)	64 (52–70)	19 M: 3 F	11.5 (10.3–12)	NP	NP	NP	1 (1–1)	NP	27 (24–31)	Yes ^4^	156 (103–188)	709 (530–786)	2400 (2000–3900)	227 (175–324)
		14	ICU COVID-	49 (36–57)	9 M: 5 F	15 (13.3–15)	NP	NP	NP	3 (1–4)	NP	26 (22–32)	NP	274 (160–328)	598 (502–645)	11,300 (4100–31,000)	175 (113–347)
Roh et al. (USA) [36]	ROTEM delta	30	ICU	63 ± 12	15 M: 15 F	NP	NP	NP	NP	NP	NP	33 ± 8.1	Yes ^1^	NP	NP	11,400 ± 7300	255 ± 103
Kong et al. (United Kingdom) [37]	ROTEM delta	1	ICU	48	F	NP	NP	NP	NP	NP	NP	28.3	Yes ^1^	196	840	510	307
		1	ICU	68	M	NP	NP	NP	NP	NP	NP	27.1	Yes ^4^	336	680	>20,000	126
Raval et al. (USA) [38]	ROTEM delta	1	ICU	63	M	NP	NP	NP	NP	NP	NP	NP	NP	NP	NP	2143	NP
Nougier et al. (France) [39]	Modified ROTEM delta (TEM-tPA)	40	ICU (ROTEM *n* = 19)	62.8 ± 13.1	NP	5.4 ± 3.1	NP	37.9 ± 13	NP	NP	NP	29 ± 5.5	NP	NP	610 ± 190	3456 ± 2641	NP
		38	IMW (ROTEM *n* = 4)	60.2 ± 14.6	NP	/	/	/	/	/	/	26.2 ± 4.8		NP	560 ± 170	874 ± 539	NP
Weiss et al. (France) [40]	Modified ROTEM delta (TEM-tPA)	5	ICU	57 ± 15	5 M: 0 F	9 ± 2	NP	NP	NP	NP	NP	NP	NP	NP	740 ± 240	1975 ± 1623	440 ± 270
Almskog et al. (Sweden) [41]	ROTEM sigma	20	ICU	62 (55–66)	12 M: 8 F	NP	NP	NP	NP	NP	NP	28 (25–32)	Yes ^5^	NP	680 (480–760)	1500 (700–4000)	252 (206–341)
		40	IMW	61 (51–74)	28 M: 12 F	/	/	/	/	/	/	26 (24–32)		NP	540 (430–650)	600 (500–1000)	212 (175–259)
Collett et al. (Australia) [42]	ROTEM sigma	6	ICU	69 (64.2–73)	5 M: 1 F	7.5 (6.25–11.75)	75.5 (65.75–105.5)	NP	NP	NP	NP	NP	NP	NP	750 (721–808)	6100 (2585–9660)	291 (213–338)
Ibañez et al. (Spain) [43]	ROTEM sigma	19	ICU	61 (55–73)	10 M: 9 F	4 (2–6)	NP	NP	NP	1 (0–3)	1.8 (0.9)	28 (27–32)	Yes ^10^	NP	620 (480–760)	1000 (600–4200)	236 (136–364)
Kruse et al. (Germany) [44]	ROTEM sigma	40	ICU	67 (57.3–76.6)	35 M: 5 F	9 (6.3–11.8)	28 (22–33)	NP	NP	NP	3 (2–4)	28.1 (24.8–32.8)	Yes ^10^	124 (84–217)	667 (470–770)	3950 (2600–5900)	194 (131–316)
Pavoni et al. (Italy) [45]	ROTEM sigma	20	ICU COVID-19 pneumonia	60.3 ± 15.2	11 M: 9 F	4.4 ± 0.8	NP	NP	NP	NP	NP	28.4 ± 4.7	Yes ^4^	NP	698 ± 8	1364 ± 965	289 ± 155
		25	ICU non COVID-19 pneumonia	66.5 ± 18.8	10 M: 15 F	2.8 ± 1.1	NP	NP	NP	NP	NP	25.2 ± 2.3		NP	349 ± 81	1476 ± 770	183 ± 70
Spiezia et al. (Italy) [46]	ROTEM sigma	56	IMW COVID-19 pneumonia	64 ± 15	37 M: 19 F	2 ± 1	NP	NP	NP	NP	NP	30 ± 4	Yes ^4^	60 ± 56	451 ± 168	1079 ± 666	277 ± 131
		56	IMW non COVID-19 pneumonia	76 ± 11	35 M: 21 F	3 ± 1	NP	NP	NP	NP	NP	27 ± 6		114 ± 77	488 ± 198	1296 ± 8	274 ± 89
Van der Linden et al. (Sweden) [47]	ROTEM sigma	12	ICU before enhanced anticoagulation	54 ± 9	12 M: 0 F	NP	NP	NP	NP	NP	NP	30.3 ± 5.6	Yes ^1^	258 (135–348)	870 ± 200	6900 (5700–10,000)	393 ± 151
		14	ICU after enhanced anticoagulation	59 ± 8	14 M: 0 F	NP	NP	NP	NP	NP	NP	28.2 ± 4.2		57 (37–137)	630 ± 250	3900 (2200–6800)	320 ± 93
Blasi et al. (Spain) [48]	ROTEM sigma	12	ICU	69 (57–76)	6 M: 6 F	5.5 (3.3–7.8)	15.5 (12–17.8)	NP	NP	NP	NP	32 (27–35)	Yes ^1^	0.77 (0.42–2.59)	393 (300–488)	2535 (860–7848)	196 (127–293)
		11	IMW	58 (42–74)	8 M: 3 F	/	/	/	/	/	/	29 (27–31)		3.28 (2.33–8.96)	502 (172–552)	565 (425–2188)	167 (154–239)
Van Veenendaal et al. (The Netherlands) [49]	ROTEM sigma	47	ICU	63 (29–79)	38 M: 9 F	/	/	42 (17–70)	/	/	/	28.8 (24.4–48.4)	Yes ^4^	NP	720 ± 160	NP	404 ± 154
Lazar et al. (USA) [50]	ROTEM sigma	1	IMW	NP	NP	/	/	/	/	/	/	NP	NP	NP	653	760	NP
		1	IMW	NP	NP	/	/	/	/	/	/	NP	NP	NP	820	1330	NP
Wright et al. (USA) [51]	TEG (NS)	44	ICU	54 (42–59)	28 M: 16 F	NP	NP	NP	NP	NP	NP	30 (27–37)	Yes ^5^	NP	656 (560–779)	1840 (935–4085)	232 (186–298)
Panigada et al. (Italy) [52]	TEG5000	24	ICU	56 (23–71)	NP	NP	NP	NP	NP	NP	NP	NP	NP	161 (39–342)	680 (234–1344)	4877 (1197–16,954)	348 (59–577)
Cordier et al. France) [53]	TEG5000	24	ICU	69 (61–71)	16 M: 8 F	NP	NP	45 (33–53)	NP	3 (2–3)	NP	28.5 (25.7–31)	NP	128 (101–249)	680 (620–790)	3600 (1960–6490)	220 (173–294)
Hightower et al. (USA) [54]	TEG5000	5	ICU	59 (38–69.5)	3 M: 2 F	NP	NP	NP	NP	NP	NP	34.4 ± 3.9	Yes ^6^	NP	658 ± 93	10,672 ± 7907	243 ± 35
Maatman et al. (USA) [55]	TEG5000	109	ICU (TEG *n* = 12)	61 ± 16	62 M: 47 F	NP	NP	NP	NP	NP	NP	34.8 ± 11.8	Yes ^5^	146 (101–227)	535 (435–651)	506 (321–973)	207 (152–255)
Mortus et al. (USA) [56]	TEG5000	21	ICU	68 ± 11	12 M: 9 F	NP	NP	NP	NP	NP	NP	NP	Yes (NS)	NP	740 ± 240	8300 ± 7000	210 ± 100
Sadd et al. (USA) [57]	TEG5000	10	ICU	58 (49–70)	8 M: 2 F	4 (3–5)	NP	NP	NP	NP	NP	35 (30–39)	Yes ^3^	20 (13–25)	676 (543–769)	3150 (1000–6620)	291 (224–408)
Yuriditsky et al. (USA) [58]	TEG5000	64	ICU	64 (57–71)	46 M: 18 F	NP	NP	NP	NP	NP	NP	NP	Yes ^7^	104 (35–158)	669 (451–838)	2374 (923–4820)	244 (176–321)
Bocci et al. (Italy) [59]	TEG6s	40	ICU	67.5 (55–77)	29M: 11F	5 ± 2.9	NP	NP	NP	2.9 ± 0.6	NP	NP	Yes ^8^	160 (75–193)	513 (304–605)	1753 (699–4435)	194 (163–281)
Stattin et al. (Sweden) [60]	TEG6s	31	ICU	65 (51–70)	25 M: 6 F	NP	NP	NP	53 (48–60)	NP	NP	30 (27–33)	Yes ^5^	214 (152–294)	NP	2100 (900–3200)	227 (163–248)
Vlot et al. (The Netherlands) [61]	TEG6s	16	ICU	67 (56–73)	12 M: 4 F	NP	NP	NP	NP	NP	NP	NP	Yes ^6^	NP	620 (590–690)	4425 (1870–5781)	347 (302–462)
Patel et al. (United Kingdom) [62]	TEG6s	39	ICU	52.5 (29–79)	32 M: 7 F	8 ± 2.5	18.7 ± 5	NP	NP	NP	NP	31.3 ± 6.1	Yes ^5^	305 ± 101	660 ± 190	6440 ± 10,434	272 ± 77
Salem et al. (United Arab Emirates) [63]	TEG6s	52	ICU	53 (39–62)	51 M: 1 F	NP	NP	NP	NP	NP	NP	25.8 (23–29.5)	Yes ^9^	50 (9–117)	400 (270–600)	4000 (3300–4000)	228 (137–292)
Shah et al. (United Kingdom) [64]	TEG6s	187	ICU (TEG *n* = 20)	57 (49–64)	124 M: 63 F	NP	13 (10–13)	NP	NP	NP	NP	28 (25–32)	Yes ^10^	202 (128–294)	700 (600–1000)	2587 (950–10,000)	241 (186–318)
Fan et al. (Singapore) [65]	TEG6s	1	IMW	39	M	NP	NP	NP	NP	NP	NP	NP	NP	136	770	2,55	NP
Masi et al. (France) [66]	Quantra	17	ICU COVID+	48 (42–58)	12 M: 5 F	12 (9–17)	NP	52 (43–63)	NP	0 (0)	NP	31 (28.8–40.5)	Yes ^3^	136 (92–315)	710 (490–790)	8390 (5330–11,180)	231 (160–245)
		11	ICU COVID-	34 (28–55)	7 M: 4 F	9 (7–17)	NP	57 (37–81)	NP	4 (36)	NP	29.3 (26–35)	NP	320 (159–367)	810 (640–945)	4640 (3200–20,000)	262 (224–334)
Ranucci et al. (Italy) [67]	Quantra	16	ICU	61 (55–65)	15 M: 1 F	NP	NP	NP	NP	NP	NP	26.4 (23.9–35.1)	Yes ^4^	NP	794 (583–933)	3500 (2500–6500)	271 (192–302)
Bachler et al. (Austria) [24]	ClotPro	20	ICU	61.5 (56.25–68)	14 M: 6 F	6.5 (3–8.25)	NP	NP	56 (53–64)	NP	NP	28.8 (24.3–31)	Yes ^1^	187.1 (116.4–275.7)	600 (553–677.25)	1554 (1227–9088)	230 (202.5–297.25)
Zátroch et al. (Hungary) [68]	ClotPro	1	ICU	62	M	NP	NP	NP	NP	NP	NP	NP	Yes ^2^	21	NP	NP	NP
		1		80	M	NP	NP	NP	NP	NP	NP	NP		176–221	448	7370	NP
		1		84	F	NP	NP	NP	NP	NP	NP	NP		230–376	544	10,600	NP

Values in italics and in brackets are the reference values; we have indicated our reference ranges * for information purposes. Comorbidities: ^1^ Overweight and obesity, associated with high blood pressure, diabetes and cardiovascular risk factors; ^2^ High blood pressure, diabetes and some additional comorbidities; ^3^ Overweight and obesity, with some additional comorbidities; ^4^ Overweight and obesity; ^5^ Overweight and obesity, associated with high blood pressure, diabetes, pulmonary disease and cardiovascular risk factors; ^6^ Overweight and obesity, associated with high blood pressure; ^7^ Overweight and obesity, associated with cardiovascular risk factors, pulmonary disease and kidney disease; ^8^ Overweight and obesity, associated with diabetes, cardiovascular risk factors, pulmonary disease and kidney disease; ^9^ Overweight and obesity, associated with high blood pressure, diabetes, kidney disease and cardiovascular risk factors; ^10^ Overweight and obesity, associated with high blood pressure, diabetes, pulmonary disease, kidney disease and cardiovascular risk factors; ^11^ Overweight and obesity, associated with high blood pressure and diabetes. Abbreviations: ICU: Intensive care unit (adults); IMW: Internal medicine ward; PICU: Pediatric intensive care unit; PW: Pediatric ward; IMV: Invasive mechanical ventilation; ECMO: Extracorporeal membrane oxygenation; RRT: Renal replacement therapy; M: Male; F: Female; SOFA score: Sequential organ failure assessment score; APACHE score: Acute physiology and chronic health evaluation score; SAPS score: Simplified acute physiology score; DIC score: Disseminated intravascular coagulation score; SIC score: Sepsis-induced coagulopathy score; BMI: Body mass index; CRP: C-reactive protein; NP: Not provided; TEG: Thromboelastography; ROTEM: Rotational thromboelastometry; TEM: Thromboelastometry; tPA: tissue plasminogen activator.

**Table 5:** Main findings of studies reporting ROTEM results (except APTEM and TEM-tPA assays).

First Author (Country)	Design	*n*	Ward	Device	Controls	EXTEM								INTEM						FIBTEM							Conclusions of the Study	Association with the Occurrence of Thrombotic Events	Definition of Hypercoagulability Assessed by VET According to the Authors
						CT (s)	CFT (s)	α Angle (°)	A(x) (mm)	MCF (mm)	ML (%)	LI30 (%)	LI60 (%)	CT (s)	CFT (s)	α Angle (°)	A(x) (mm)	MCF (mm)	ML (%)	CT (s)	CFT (s)	A(x) (mm)	MCF (mm)	ML (%)	LI30 (%)	LI60 (%)			
Iwasaki et al. (Japan) [26]	Case report	1	ICU (T1: D0)	NS	Reference range as assessed by the manufacturer	N	N	NP	↑	↑	NP	100	N	N	N	NP	↑	↑	NP	N	↓	↑	↑	NP	100	100	Hypercoagulable state not detected by conventional coagulation tests	NA	Increased MCF and decreased CFT
			ICU (T2: D1)			N	N	NP	↑	↑	NP	100	N	N	N	NP	↑	↑	NP	N	↓	↑	↑	NP	100	100			
			ICU (T3: D2)			N	N	NP	↑	↑	NP	100	N	N	N	NP	↑	↑	NP	N	↓	↑	↑	NP	100	100			
Pavoni et al. (Italy) [27]	Retrospective observational study	40	ICU (T1: upon admission)	ROTEM gamma	Reference range as assessed by the manufacturer	N-↑ ^1^	N-↓ ^1^	NP	↑ ^1^	↑ ^1^	NP	NP	N^1^	N^1^	N-↓ ^1^	NP	↑ ^1^	↑ ^1^	NP	NP	NP	NP	From ↑ to N ^2^	NP	NP	NP	Inflammatory state associated with a hypercoagulable state rather than a consumption coagulopathy	NA	Increased MCF and decreased CFT
		40	ICU (T2: 5 days later)																										
		33/40	ICU (T3: 10 days later)																										
Boscolo et al. (Italy) [28]	Prospective observational study	32	ICU	ROTEM delta	Reference range previously established in healthy adults	N	N	NP	NP	N	NP	NP	NP	N	N	NP	NP	N	NP	NP	NP	NP	↑ ^3^	NP	NP	NP	Hypercoagulable state assessed by an increased MCF in FIBTEM. No differences between patients with and without TE	No	Increased MCF
		32	IMW			N	N			N				N	N			N											
Corrêa et al. (Brazil) [29]	Prospective observational study	30	ICU	ROTEM delta	Reference range as assessed by the manufacturer	N-↑	N	NP	NP	↑	N	NP	NP	N	N	NP	NP	↑	N	NP	NP	NP	↑	NP	NP	NP	Hypercoagulable state with increased MCF related to high fibrinogen levels	NA	Decreased CT and/or CFT in EXTEM and/or INTEM, and/or increased MCF in EXTEM, INTEM and/or FIBTEM
		16/30	SOFA score < 10			N-↑	N	NP	NP	↑	N	NP	NP	N	N	NP	NP	↑	N	NP	NP	NP	↑	NP	NP	NP			
		14/30	SOFA score > 10			N-↑	N	NP	NP	↑	N	NP	NP	N	N	NP	NP	↑	↓	NP	NP	NP	↑	NP	NP	NP			
Madathil et al. (USA) [30]	Prospective observational study	5/11	D-dimers levels ≤ 3245 µg/L	ROTEM delta	Reference range as assessed by the manufacturer	N	NP	NP	N-↑	NP	0	NP	NP	NP	NP	NP	NP	NP	NP	NP	NP	NP	↑	NP	NP	NP	Critically ill COVID patients have significant elevation in D-dimers levels consistent with microthrombosis and an impaired systemic fibrinolysis	NA	NP
		6/11	D-dimers levels > 3245 µg/L			N	NP	NP	N-↑	NP	0	NP	NP	NP	NP	NP	NP	NP	NP	NP	NP	NP	↑	NP	NP	NP			
Spiezia et al. (Italy) [31]	Prospective observational case control study	22	ICU	ROTEM delta	Reference range previously established in healthy adults	N	↓	NP	NP	↑	N	NP	NP	N	↓	NP	NP	↑	N	NP	NP	NP	↑	NP	NP	NP	Hypercoagulable state rather than a consumptive coagulopathy such as DIC, due to both increased levels of fibrinogen and excessive fibrin polymerization	NA	Increased MCF and decreased CFT
Tsantes et al. (Greece) [32]	Prospective observational study	11	ICU COVID-19 patients	ROTEM delta	Reference range previously established in healthy adults	N	↓	↑	↑	↑	↓	NP	↑	NP	NP	NP	NP	NP	NP	NP	NP	NP	NP	NP	NP	NP	Hypercoagulable state and hypofibrinolytic profile with decreased CFT and ML, and increased aα angle, A10, MCF and LI60. More pronounced trend in ICU patients	NA	Increased clot amplitude (A(x) and/or MCF)
		9	ICU non-COVID-19 patients			N	↓	↑	↑	↑	↓	NP	↑	NP	NP	NP	NP	NP	NP	NP	NP	NP	NP	NP	NP	NP			
		21	IMW COVID-19 patients			↑	↓	↑	↑	↑	N	NP	↑	NP	NP	NP	NP	NP	NP	NP	NP	NP	NP	NP	NP	NP			
Al-Ghafry et al. (USA) [33]	Retrospective observational study	8	Pediatric COVID-19 patients (5 PICU, 3 PW)	ROTEM delta	Reference range according to age	2/8 ↑	1/8 ↓	NP	2/8 ↑	4/8 ↑	NP	NP	NP	1/8 ↓	1/8 ↓	NP	2/8 ↑	3/8 ↑	NP	NP	NP	6/8 ↑	6/8 ↑	NP	NP	NP	Hypercoagulable state comparable to adults. No correlation between MCF and Clauss fibrinogen nor D-dimers levels	No	Increased clot amplitude (A(x) and/or MCF)
Creel-Bulos et al. (USA) [34]	Retrospective observational study	25	ICU	ROTEM delta	Reference range as assessed by the manufacturer	NP	NP	NP	NP	↑	↓	NP	NP	NP	NP	NP	NP	NP	NP	NP	NP	NP	↑	NP	NP	NP	Impaired fibrinolysis (fibrinolysis shutdown) is associated with a higher rate of TE	Yes	NP
Hoechter et al. (Germany) [35]	Retrospective observational case control study	22 (ROTEM *n* = 11)	ICU COVID-19 patients	ROTEM delta	Reference range as assessed by the manufacturer	N	N	NP	NP	N	N	NP	NP	NP	NP	NP	NP	NP	NP	NP	NP	NP	↑	NP	NP	NP	COVID-19 patients have higher coagulatory potential	No	NP
		14	ICU non-COVID-19 patients			N	N	NP	NP	N	N	NP	NP	NP	NP	NP	NP	NP	NP	NP	NP	NP	N	NP	NP	NP			
Roh et al. (USA) [36]	Retrospective observational case control study	30	ICU COVID-19 ARDS patients	ROTEM delta	Surgical non COVID patients	↑	NP	NP	NP	↑	NP	NP	NP	↑	NP	NP	NP	↑	NP	NP	NP	NP	↑	NP	NP	NP	Critically-ill COVID-19 patients characterized by elevated D-dimers levels and hypercoagulable state related to increased fibrinogen. Negative correlation between D-dimers levels and ROTEM MCF	NA	Increased MCF two SD above normal healthy control testing
		30	ICU surgical non-COVID-19 patients																										
Kong et al. (United Kingdom) [37]	Case report	1	ICU	ROTEM delta	Reference range as assessed by the manufacturer	↑	N	N	↑	↑	N	NP	N	NP	NP	NP	NP	NP	NP	↑	N	↑	↑	N	NP	↑	Hypercoagulable state with decreased CFT and increased MCF	NA	Increased MCF
		1	ICU			↑	↑	↓	↓	↓	↓	NP	↑	NP	NP	NP	NP	NP	NP	↑	NP	N	N	↓	NP	↑	Hypocoagulable state with increased CFT and decreased MCF, with fibrinolysis shutdown as assessed by decreased ML%, increased LI60 and high level of D-dimers		
Raval et al. (USA) [38]	Case report	1	ICU	ROTEM delta	Reference range as assessed by the manufacturer	NP	↓	↑	NP	↑	NP	NP	NP	NP	NP	NP	NP	↑	NP	NP	NP	NP	↑	NP	NP	NP	Hypercoagulable state: VET as a possible screening tool for severe disease?	NA	Increased MCF and α angle, and decreased CFT
Weiss et al. (France) [40]	Prospective observational case control study	5	ICU	Modified ROTEM delta (TEM-tPA)	Reference range established in healthy adults	NP	NP	NP	NP	↑	NP	NP	NP	NP	NP	NP	NP	NP	NP	NP	NP	NP	↑	NP	NP	NP	No clot lysis after 60 min in patients as compared to healthy controls. Resistance to clot lysis not only related to high fibrinogen levels: dysregulation of the fibrinolytic system?	NA	Increased MCF
Almskog et al. (Sweden) [41]	Prospective observational study	20	ICU	ROTEM sigma	Reference range previously established in healthy adults	↑	↓	NP	↑ ^4^	↑ ^3^	NP	100	NP	↑	↓	NP	↑ ^4^	↑ ^3^	NP	NP	NP	NP	↑ ^3^	NP	NP	NP	Association between MCF-FIBTEM and Clauss fibrinogen. Hypercoagulable state as assessed by ROTEM can be seen early after admission, with a more pronounced pattern in patients with increased disease severity: ROTEM useful to predict TE and care level?	NA	Increased MCF
		40	IMW			↑	↓	NP	↑	↑	NP	100	NP	↑	N	NP	↑	↑	NP	NP	NP	NP	↑	NP	NP	NP			
Collett et al. (Australia) [42]	Prospective observational study	6	ICU	ROTEM sigma	Reference range as assessed by the manufacturer	NP	N-↓ 2/6	NP	5/6 ↑	↑ 5/6	N 6/6	NP	NP	NP	↓ 5/6	NP	NP	↑ 5/6	0	NP	NP	6/6 ↑	↑ 6/6	0	NP	NP	Hypercoagulable state as assessed by VET with increased MCF, minimal fibrinolysis and hyperfibrinogenemia	NA	Increased clot amplitude (A(x) and/or MCF)
Ibañez et al. (Spain) [43]	Prospective observational study	19	ICU	ROTEM sigma	Reference range as assessed by the manufacturer	N-↑	N-↓	NP	NP	↑	NP	100	100	N	N	NP	NP	N	NP	NP	NP	NP	↑	NP	100	100	Hypercoagulable state mainly characterized by decreased fibrinolytic capacity associated with a paradoxical increase in D-dimers levels: fibrinolysis shutdown?	NA	Increased MCF
Kruse et al. (Germany) [44]	Prospective observational study	40	ICU	ROTEM sigma	Reference range as assessed by the manufacturer	↑	↓	NP	NP	↑	↓	NP	NP	↑	↓	NP	NP	↑	↓	N	N	NP	↑	NP	NP	NP	Hypercoagulable state with increased MCF related to high fibrinogen levels. Hypofibrinolysis with decreased ML%. Combination of ML% with D-dimers levels revealed high sensitivity and specificity of TE risk prediction	Yes	NP
		23/40	≥1 TE			↑	↓	NP	NP	↑	↓ ^5^	NP	NP	↑ ^6^	↓	NP	NP	↑	↓ ^5^	N	N	NP	↑	NP	NP	NP			
		17/40	no TE			↑	↓			↑	N			↑	↓			↑	N	N	N		↑						
Pavoni et al. (Italy) [45]	Prospective case controls observational study	20	ICU COVID-19 pneumonia (T1: upon admission)	ROTEM sigma	Reference range as assessed by the manufacturer	N	↓	NP	↑ ^7^	↑ ^8^	N	NP	NP	N	↓	NP	↑ ^9^	↑ ^10^	N	NP	NP	NP	↑ ^8^	NP	NP	NP	Hypercoagulable state with decreased CFT and increased MCF, more pronounced in patients with COVID-19 pneumonia	NA	Increased MCF
		25	ICU non-COVID-19 pneumonia (T1: upon admission)			N	N		N	N	N			N	N		N	N	N				N						
		20	ICU COVID-19 pneumonia (T2: 10 days later)			N	↓	NP	↑ ^7^	↑ ^8^	N	NP	NP	N	N	NP	N	N	N	NP	NP	NP	N	NP	NP	NP			
		25	ICU non-COVID-19 pneumonia (T2: 10 days later)			N	N		N	N	N			N	N		N	N	N				N						
Spiezia et al. (Italy) [46]	Prospective case controls observational study	56	IMW COVID-19 pneumonia	ROTEM sigma	Healthy adult volunteers age- and sex-matched	N	↓ ^11^	NP	NP	↑ ^12^	N	NP	NP	N	↓ ^11^	NP	NP	↑ ^12^	N	NP	NP	NP	↑ ^12^	NP	NP	NP	Hypercoagulable state with decreased CFT and increased MCF, more pronounced in patients with COVID-19 pneumonia	NA	Decreased CFT and increased MCF
		56	IMW non-COVID-19 pneumonia																										
Van der Linden et al. (Sweden) [47]	Cross-sectional cohort study	12	ICU before enhanced anticoagulation	ROTEM sigma	Reference range previously established in healthy adults	N	NP	NP	NP	↑	NP	NP	NP	N	NP	NP	NP	↑	NP	↑	NP	NP	↑ ^13^	NP	NP	NP	A more aggressive anticoagulation is associated with a reduction in FIBTEM-MCF (*p* < 0.001), in Clauss fibrinogen (*p* < 0.05), in inflammatory biomarkers and in pulmonary embolism outcome (*p* < 0.05)	NA	Increased MCF
		14	ICU after enhanced anticoagulation																										
Blasi et al. (Spain) [48]	Retrospective observational study	12	ICU	ROTEM sigma	Reference range as assessed by the manufacturer	N	NP	NP	NP	N-↑	NP	NP	100	N-↑	NP	NP	NP	N-↑	NP	NP	NP	NP	N-↑	NP	NP	NP	Hypercoagulable state more pronounced in sicker patients and related to hyperfibrinogenemia and low fibrinolysis despite anticoagulation	NA	Increased MCF
		11	IMW										N	N															
Van Veenendaal et al. (The Netherlands) [49]	Retrospective observational study	47	ICU	ROTEM sigma	Reference range as assessed by the manufacturer	↑	N-↓	NP	↑	↑	NP	NP	NP	N	↓	NP	↑	↑	NP	NP	NP	NP	↑	NP	NP	NP	Hypercoagulable state with decreased CFT and increased MCF related to high fibrinogen levels. Correlation between increased CT and prolonged aPTT and PT	No	Decreased CFT and increased MCF
		10/47	≥1 TE			↑	N	NP	↑ ^14^	↑ ^15^	NP	NP	NP	N	↓ ^16^	NP	↑ ^15^	↑	NP	NP	NP	NP	↑	NP	NP	NP			
		37/47	no TE			↑	↓							N				↑					↑						
Lazar et al. (USA) [50]	Case report	1	IMW	ROTEM sigma	Local reference range	N	↓	↑	↑	↑	N	NP	NP	N	N	N	↑	↑	N	N	NP	↑	↑	N	NP	NP	Hypercoagulable state present early in the clinical course of the disease	NA	Increased MCF
		1	IMW			↑	N	N	N-↑	↑	N	NP	NP	↑	N	N	N	N	N	N	NP	↑	↑	N	NP	NP			

^1^ No difference between D0 and D10 (*p* > 0.05); ^2^ Normalization between D0 and D10 (*p* < 0.05); ^3^ Higher MCF in ICU patients than in IMW ones (*p* < 0.05); ^4^ Higher A(x) in ICU patients than in IMW ones (*p* < 0.05); ^5^ Lower ML in patients with TE (*p* < 0.05); ^6^ Longer CT in patients with TE (*p* < 0.05); ^7^ Higher clot amplitude in COVID-19 patients upon admission (*p* < 0.0001); ^8^ Higher MCF in COVID-19 patients upon admission (*p* < 0.0001); ^9^ Higher clot amplitude in COVID-19 patients upon admission (*p* < 0.05); ^10^ Higher MCF in COVID-19 patients upon admission (*p* < 0.05); ^11^ Shorter CFT in COVID-19 patients (*p* < 0.001); ^12^ Higher MCF in COVID-19 patients (*p* < 0.05); ^13^ Higher MCF with low dose of LMWH (*p* < 0.001); ^14^ Higher A(x) in patients with TE (*p* < 0.05); ^15^ Higher MCF in patients with TE (*p* < 0.05); ^16^ Shorter CFT in patients with TE (*p* < 0.05). Results from the APTEM assay were only reported by one case report [26] and were consistent with the absence of hyperfibrinolysis. Results from the HEPTEAM assay were reported by only four studies and are displayed apart [26,41,44,50]. Results from the investigator-modified assay derived from EXTEM assay to investigate potential hypofibrinolysis (TEM-tPA) were reported by only two studies and are displayed apart [39,40]. Abbreviations: ICU: Intensive care unit (adults); IMW: Internal medicine ward; PICU: Pediatric intensive care unit; PW: Pediatric ward; TE: Thrombotic events; N: Result within the reference range; ↑: Result above the reference range; ↓: Result below the reference range; N-↑: Result at the upper limit of the reference range; N-↓: Result at the lower limit of the reference range; NP: Not provided; NA: Not assessed; tPA: tissue plasminogen activator.

**Table 6:** Main findings of studies reporting results from the HEPTEM assay (ROTEM).

First Author (Country)	Design	*n*	Ward	Device	Controls	INTEM						HEPTEM					Conclusions of the Study	Association with the Occurrence of Thrombotic Events	Definition of Hypercoagulability Assessed by VET According to the Authors
						CT (s)	CFT (s)	α Angle (°)	A(x) (mm)	MCF (mm)	ML (%)	CT (s)	CFT (s)	α Angle (°)	MCF (mm)	ML (%)			
Iwasaki et al. (Japan) [26]	Case report	1	ICU (T1: D0)	NS	Reference range as assessed by the manufacturer	N	N	NP	↑	↑	NP	NP	NP	NP	NP	NP	Hypercoagulable state not detected by conventional coagulation tests	NA	Increased MCF and decreased CFT
			ICU (T2: D1)			N	N	NP	↑	↑	NP	N	N	NP	N	NP			
			ICU (T3: D2)			N	N	NP	↑	↑	NP	N	N	NP	N	NP			
Almskog et al. (Sweden) [41]	Prospective observational study	20	ICU	ROTEM sigma	Reference range previously established in healthy adults	↑	↓	NP	↑ ^4^	↑ ^3^	NP	↑	NP	NP	NP	NP	Association between MCF-FIBTEM and Clauss fibrinogen. Hypercoagulable state as assessed by ROTEM can be seen early after admission, with a more pronounced pattern in patients with increased disease severity: ROTEM useful to predict TE and care level?	NA	Increased MCF
		40	IMW			↑	N	NP	↑	↑	NP	↑	NP	NP	NP	NP			
Kruse et al. (Germany) [44]	Prospective observational study	40	ICU	ROTEM sigma	Reference range as assessed by the manufacturer	↑	↓	NP	NP	↑	↓	N	↓	NP	↑	NP	Hypercoagulable state with increased MCF related to high fibrinogen levels. Hypofibrinolysis with decreased ML%. Combination of ML% with D-dimers levels revealed high sensitivity and specificity of TE risk prediction	Yes	NP
		23/40	≥1 TE			↑	↓	NP	NP	↑	↓	N	↓	NP	↑	NP			
		17/40	no TE			↑	↓			↑	N	N			↑				
Lazar et al. (USA) [50]	Case report	1	IMW	ROTEM sigma	Local reference range	N	N	N	↑	↑	N	N	N	N	↑	N	Hypercoagulable state present early in the clinical course of the disease	NA	Increased MCF
		1	IMW			↑	N	N	N	N	N	N	N	N	N	N			

Abbreviations: ICU: Intensive care unit (adults); IMW: Internal medicine ward; TE: Thrombotic events; N: Result within the reference range; ↑: Result above the reference range; ↓: Result below the reference range; NP: Not provided; NA: Not assessed; tPA: tissue plasminogen activator.

**Table 7:** Main findings of studies reporting results from the TEM-tPA assay (ROTEM).

First author (Country)	Design	*n*	Ward	Device	Controls	EXTEM Assay							TEM-tPA Assay				Conclusions	Association with the Occurrence of Thrombotic Events Outcomes	Definition of Hypercoagulability Assessed by VET According to the Authors
						CT (s)	CFT (s)	α angle (°)	A(x) (mm)	MCF (mm)	ML (%)	LI30 (%)	LI60 (%)	MCF (mm)	LI30 (%)	ML (%)			
Nougier et al. (France) [39]	Prospective observational case control study	19	ICU	Modified ROTEM delta (TEM-tPA)	Reference range previously established in healthy adults	NP	NP	NP	NP	NP	NP	NP	NP	↑ ^1^	↑ ^2^	NP	Hypercoagulable state associated with impaired fibrinolysis leading to a high thrombin generation despite adequate antithrombotic therapy	NA	Increased MCF
		4	IMW																
Weiss et al. (France) [40]	Prospective observational case control study	5	ICU	Modified ROTEM delta (TEM-tPA)	Reference range established in healthy adults	NP	NP	NP	NP	↑	NP	NP	NP	↑	NP	↓	No clot lysis after 60 min in patients as compared to healthy controls. Resistance to clot lysis not only related to high fibrinogen levels: dysregulation of the fibrinolytic system?	NA	Increased MCF

^1^ No difference between the two groups (*p* > 0.05); ^2^ Higher LI30 in COVID-19 patients (*p* < 0.05). Abbreviations: ICU: Intensive care unit (adults); IMW: Internal medicine ward; N: Result within the reference range; ↑: Result above the reference range; ↓: Result below the reference range; NP: Not provided; NA: Not assessed; tPA: tissue plasminogen activator.

**Table 8:** Main findings of studies reporting TEG results.

First Author (Country)	Design	*n*	Ward	Device	Controls	CRT Assay /Rapid-TEG							CK Assay					CKH Assay					CFF Assay		Conclusions of the Study	Association with the Occurrence of Thrombotic Events	Definition of Hypercoagulability Assessed by VET According to the Authors
						TEG-ACT	R (min)	K (min)	α angle (°)	A10 (mm)	MA (mm)	LY30 (%)	R (min)	K (min)	α angle (°)	MA (mm)	LY30 (%)	R (min)	K (min)	α angle (°)	MA (mm)	LY30 (%)	A10 (mm)	MA (mm)			
Wright et al. (USA) [51]	Retrospective observational study	44	ICU	NP	Reference range as assessed by the manufacturer	NP	NP	NP	NP	NP	NP	NP	NP	NP	NP	NP	NP	N	NP	N	↑	0	NP	NP	Fibrinolysis shutdown, as evidenced by elevated D-dimers levels and complete failure of clot lysis at 30 min on thromboelastography predicts thromboembolic events and need for hemodialysis in critically ill patients with COVID-19.	Yes: higher rate of TE (*p* < 0.05), shorter time to TE (*p* = 0.001)	Increased MA despite appropriate prophylactic anticoagulation
Panigada et al. (Italy) [52]	Prospective observational study	24	ICU	TEG5000	Reference range previously established in healthy adults	NP	NP	NP	NP	NP	NP	NP	NP	NP	NP	NP	NP	12/24 ↓	22/24 ↓	18/24 ↑	21/24 ↑	24/24 ↓	NP	NP	Hypercoagulable state assessed by a shortened K, decrease LI30 and increase MA and α angle	NA	Decreased R, K or LY30 as well as increased α angle or MA
Cordier et al. (France) [53]	Retrospective observational study	24	ICU (T1: upon admission)	TEG5000	Reference range previously established in healthy adults	NP	NP	NP	NP	NP	NP	NP	NP	NP	NP	NP	NP	↓ ^1^	↓ ^1^	↑ ^1^	↑ ^2^	0	NP	NP	Hypercoagulable state which persists even in case of favorable clinical evolution. No difference between obese and non-obese patients. No difference between according to the severity of CT lesions. No difference between patients who developed TE and those who did not. No difference between patients who died and those who survived	No	Decreased R, K or LY30 as well as increased α angle or MA
		10/24	ICU (T2: at discharge)																								
Hightower et al. (USA) [54]	Retrospective observational study	5	ICU	TEG5000	Reference range as assessed by the manufacturer	NP	NP	NP	NP	NP	NP	NP	NP	NP	NP	NP	NP	N	N	↑	↑	0	NP	NP	Hypercoagulable state with impaired fibrinolysis	NA	Decreased R or K as well as increased α angle or MA
Maatman et al. (USA) [55]	Retrospective multi-center observational study	109 (TEG *n* = 12)	ICU	TEG5000	Reference range previously established in healthy adults	NP	NP	NP	NP	NP	NP	NP	NP	NP	NP	NP	NP	8/12 ↓	5/12 ↓	5/12 ↑	5/12 ↑	NP	NP	NP	Hypercoagulable state as assessed by a raised MA and an absent fibrinolysis, despite at least prophylactic dose of LWMH or HNF. However, no systematic association between hypercoagulable state as assessed by TEG and TE outcomes	No	At the parameters level: decreased R or K as well as increased α angle or MA. At the thromboelastography level: two or more parameters beyond one SD of the age- and gender-matched controls
		78/109	no TE			NP	NP	NP	NP	NP	NP	NP	NP	NP	NP	NP	NP	5/8 ↓	3/8 ↓	3/8 ↑	3/8 ↑	NP	NP	NP			
		31/109	≥1 TE			NP	NP	NP	NP	NP	NP	NP	NP	NP	NP	NP	NP	3/4 ↓	2/4 ↓	2/4 ↑	2/4 ↑	NP	NP	NP			
Mortus et al. (USA) [56]	Retrospective cohort study	21	ICU	TEG5000	Reference range previously established in healthy adults	NP	NP	NP	NP	NP	NP	NP	↑	NP	N	↑	N	N	NP	N-↑	↑	N	NP	NP	Innate TEG MA provides 100% sensitivity and 100% negative predictive value to discriminate between patients with high rate of TE and those with low rate.	Yes: Innate TEG MA provides 100% sensitivity and 100% negative predictive value to discriminate between patients with high rate of TE and those with low rate.	α angle > 73° and/or MA > 65 mm after heparinase correction
		11/21	≤ 1 TE			NP	NP	NP	NP	NP	NP	NP	↑	NP	N	N	N	N	NP	N-↑	↑	N	NP	NP			
		10/21	≥ 2 TE			NP	NP	NP	NP	NP	NP	NP	N-↑	NP	N	↑	N-↑	N	NP	↑	↑	N	NP	NP			
Sadd et al. (USA) [57]	Retrospective observational cohort study	10	ICU	TEG5000	Reference range as assessed by the manufacturer	NP	NP	NP	NP	NP	NP	NP	N	NP	NP	NP	NP	↓	N	↑	↑	0	NP	NP	Hypercoagulable state with impaired fibrinolysis	NA	NP
		4/10	≥1 TE and after tPA thrombolysis			NP	NP	NP	NP	NP	NP	NP	N	NP	NP	NP	NP	N	N	N-↑	N	0	NP	NP			
		6/10	No TE			NP	NP	NP	NP	NP	NP	NP	NP	NP	NP	NP	NP	NP	NP	NP	NP	NP	NP	NP			
Yuriditsky et al. (USA) [58]	Retrospective observational study	64	ICU	TEG5000	Reference range as assessed by the manufacturer	NP	NP	NP	NP	NP	NP	NP	19/64 ↑	NP	NP	NP	NP	28/64 ↓	28/64 ↓	45/64 ↑	38/64 ↑	N	NP	NP	No correlation between D-dimers levels and LY30, no association between TEG variables and TE	No	R < 5 mn, K < 1 mn, MA > 70 mm
		26/64	D-dimers levels ≤ 2000 µg/L			NP	NP	NP	NP	NP	NP	NP	N ^3^	NP	NP	NP	NP	N	N ^3^	↑ ^3^	↑ ^3^	N ^3^	NP	NP			
		38/64	D-dimers levels > 2000 µg/L															↓ ^4^									
Bocci et al. (Italy) [59]	Prospective observational study	40	ICU (T1)	TEG6s	Reference range as assessed by the manufacturer	N-↓ ^5^	N ^5^	N ^5^	↑ ^5^	↑ ^5^	↑ ^5^	0	N ^5^	N ^5^	N ^5^	NP	0	N ^5^	N ^5^	N-↑ ^5^	N-↑ ^5^	0	↑ ^5^	↑ ^5^	Hypercoagulable state as assessed by an increased α angle and clot amplitude, associated with an absent lysis of the clot at 30 min but no correlation with the occurrence of TE. No difference between D0 and D7, nor between patients who survived and those not	No	NP
		26/40	ICU (T2: 7 days later)																								
		23/40	Dead			N-↓ ^3^	N ^3^	N-↓ ^3^	↑ ^3^	↑ ^3^	N-↑ ^3^	0	N ^3^	N ^3^	N ^3^	NP	0	N ^3^	N ^3^	N-↑ ^3^	N-↑ ^3^	0	↑ ^3^	↑ ^3^			
		17/40	Alive																								
Stattin et al. (Sweden) [60]	Prospective observational study	31	ICU (T1: within 4 days after admission)	TEG6s	Reference range as assessed by the manufacturer	NP	NP	NP	NP	NP	NP	NP	N ^5^	NP	NP	NP	NP	N ^5^	NP	N-↑ ^5^	↑ ^5^	0	NP	NP	Hypercoagulable state as assessed by MA on TEG with insufficient effect of standard doses of LMWH. Neither anti-Xa levels nor TEG can reliably determine the effect of LMWH in patients with COVID-19.	No	Increased MA
		11/31	ICU (T2: between D4 and D7)																								
		11/31	ICU (T3: 7 days later)																								
		5/31	≥1 TE			NP	NP	NP	NP	NP	NP	NP	N ^3^	NP	NP	NP	NP	N ^3^	NP	N-↑ ^3^	↑ ^3^	0	NP	NP			
		26/31	No TE																								
Vlot et al. (The Netherlands) [61]	Prospective observational study	16	ICU (T1)	TEG6s	Reference range as assessed by the manufacturer	NP	NP	NP	NP	NP	NP	NP	NP	N-↓	NP	↑	NP	NP	NP	NP	NP	NP	NP	↑	Despite anti-Xa levels within the target range of pharmacodynamic endpoint, VET still demonstrates a procoagulant pattern with a clot strength dominated by the fibrinogen component	NA	NP
			ICU (T2)																								
Patel et al. (United Kingdom) [62]	Retrospective observational study	39	ICU		Reference range as assessed by the manufacturer	NP	NP	NP	NP	NP	NP	NP	NP	NP	NP	NP	NP	NP	NP	NP	21/39 ↑	0	NP	29/39 ↑	Hypercoagulable state as assessed by a raised MA and an absent fibrinolysis, despite at least prophylactic dose of LWMH or HNF	NA	Increased MA and particularly in CFF assay
Salem et al. (United Arab Emirates) [63]	Retrospective observational study	52	ICU	TEG6s	Reference range as assessed by the manufacturer	NP	NP	NP	NP	NP	NP	NP	NP	NP	NP	NP	NP	N	N	N	N	0	NP	NP	Hypercoagulable state as assessed by TEG not associated with the occurrence of TE	No	R < 4.3 min, K < 0.8 min, MA > 69 mm, α angle > 77°
		14/52	≥1 TE			NP	NP	NP	NP	NP	NP	NP	NP	NP	NP	NP	NP	N ^3^	N ^3^	N ^3^	N ^3^	0	NP	NP			
		38/52	No TE																								
		16/52	hypercoagulable profile			NP	NP	NP	NP	NP	NP	NP	NP	NP	NP	NP	NP	N ^3^	N ^3^	N-↑ ^3^	↑ ^6^	0	NP	NP			
		36/52	non hypercoagulable profile																		N						
Shah et al. (United Kingdom) [64]	Multicenter retrospective observational study	187 (TEG *n* = 20)	ICU	TEG6s	Reference range as assessed by the manufacturer	NP	NP	NP	NP	NP	NP	NP	NP	NP	NP	NP	NP	N	NP	N-↑	↑	0	NP	↑	Hypercoagulable state as assesses by VET, but with no discrimination between patients who will undergo TE and patients who won’t	No	α angle and MA ≥ the upper limit of the reference range, extremely low LY30
		81/187	≥1 TE			NP	NP	NP	NP	NP	NP	NP	NP	NP	NP	NP	NP	N ^3^	NP	N-↑ ^3^	↑ ^3^	0	NP	↑ ^3^			
		106/187	No TE																								
Fan et al. (Singapore) [65]	Case report	1	IMW	TEG6s	Reference range as assessed by the manufacturer	N	N	N	↑	NP	↑	0	N	N	↑	↑	N	N	N	↑	N-↑	NP	NP	↑	Hypercoagulable state assessed by VET with an excessive fibrinogen component to clot strength	NA	Increased MA
						N	N	↓	↑	NP	↑	N	↑	N	N	N	0	↑	N	N	↑	NP	NP	↑			

^1^*p* < 0.001 compared with healthy subjects, no difference between value at admission and at discharge (*p* > 0.05); ^2^ *p* < 0.001 compared with healthy subjects, higher MA at discharge (*p* < 0.05); ^3^ No difference between the two groups (*p* > 0.05); ^4^ *p* = 0.001 compared with patients with D-dimers levels ≤ 2000); ^5^ No difference with baseline value (*p* > 0.05). Abbreviations: ICU: Intensive care unit; IMW: Internal medicine ward; UFH: Unfractionated heparin; LMWH: Low molecular weight heparin; ECMO: Extracorporeal membrane oxygenation; RRT: Renal replacement therapy; TE: Thrombotic events; DVT: Deep vein thrombosis; PE: Pulmonary embolism; TEG: Thromboelastography; N: Result within the reference range; ↑: Result above the reference range; ↓: Result below the reference range; N-↑: Result at the upper limit of the reference range; N-↓: Result at the lower limit of the reference range; NP: Not provided; NS: Not specified; NA: Not assessed.

**Table 9:** Main findings of studies reporting Quantra results using the QPlus cartridge.

First Author (Country)	Design	*n*	Ward	Controls	Clotting Time (CT, s)	Heparinase Clotting Time (CTH, s)	Clot Time Ratio (CT/CTH)	Clot Stiffness (CS, hPA)	Fibrinogen Contribution to Clot Stiffness (FCS, hPA)	Platelet Contribution to clot Stiffness (PCS, hPA)	Conclusions of the Study	Association with the Occurrence of Thrombotic Events	Definition of Hypercoagulability Assessed by VET According to the Authors
Masi et al. (France) [66]	Prospective case control study	11/28	ICU non-COVID-19 ARDS	Reference range as assessed by the manufacturer	N	N	N	N	↑	↑	Significant increase in procoagulants leading to a pronounced imbalance between procoagulants and anticoagulants, and a subsequent uncontrolled thrombin generation. No fibrinolysis shutdown	NA	NP
		17/28	ICU COVID-19 ARDS		N	N	N	↑ ^1^	↑ ^2^	↑ ^1^			
Ranucci et al. (Italy) [67]	Prospective observational study	16 (T0: baseline)	ICU mechanically ventilated	Reference range as assessed by the manufacturer	N ^3^	NP	NP	↑	↑	↑	Procoagulant profile with a trend to normalization after an increased thromboprophylaxis	NA	NP
		9/16 (T1: 14 days later)						N ^4^	↑ ^4^	N ^4^			

^1^*p* < 0.05 as compared with ICU non-COVID-19 patients; ^2^ *p* < 0.001 as compared with ICU non-COVID-19 patients; ^3^ No difference from baseline value with >0.05; ^4^ *p* < 0.05 as compared with baseline value. Abbreviations: ICU: Intensive care unit (adults); ARDS: Acute respiratory distress syndrome; N: Result within the reference range; ↑: Result above the reference range; NP: Not provided; NA: Not assessed.

**Table 10:** Main findings of studies reporting ClotPro results.

First Author (COUNTRY)	Design	*n*	Ward	Controls	EX-Test					IN-Test					FIB-Test			tPA-Test				Conclusions	Association with the Occurrence of Thrombotic Events Outcomes	Definition of Hypercoagulability Assessed by VET According to the Authors
					CT (s)	CFT (s)	A(x) (mm)	MCF (mm)	ML (%)	CT (s)	CFT (s)	A(x) (mm)	MCF (mm)	ML (%)	CT (s)	A(x) (mm)	MCF (mm)	CT (s)	MCF (mm)	ML (%)	LT (s)			
Bachler et al. (Austria) [24]	Retrospective study	20	ICU	Reference range established in healthy adults	N	NP	↑ ^1^	↑ ^1^	N	N	NP	↑ ^1^	↑ ^1^	N	N	↑ ^1^	↑ ^1^	N	↑ ^1^	N	↑ ^1^	Hypercoagulable pattern assessed by increased clot amplitude and MCF in all assays. No difference in TE outcomes between patients with impaired fibrinolysis (assessed by a prolonged clot lysis time in tPA assay) and patients with normal clot lysis time	No	Increased MCF. Definition not relying on VET =difficulties in reaching the anti-Xa target range despite high doses of LMWH or elevated D-dimer levels > 2000 µg/L
		6/20	ICU with LT ≤ 393 s		N ^2^	NP	N	N	N	N	NP	N	N	N	N	↑	↑	N	N	N	N			
		14/20	ICU with LT > 393 s		N		↑ ^3^	↑ ^3^	↓ ^3^	N		↑ ^3^	↑ ^3^	↓ ^4^	N	↑ ^3^	↑ ^3^	N	↑ ^3^	↓ ^3^	↑ ^3^			
Zátroch et al. (Hungary) [68]	Case report	1	ICU	Reference range as assessed by the manufacturer	N	N	N	N	N	N	N	N	N	N	N	↑	↑	N	↑	N	NP	Procoagulation, hypercoagulation and fibrinolysis shutdown	NA	Procoagulability: decreased CT. Hypercoagulability: Increased MCF
		1	ICU		↑	N	↑	↑	N	N	↓	↑	↑	N	↑	↑	↑	N	↑	N	NP			
		1	ICU		↑	N	↑	↑	NP	↑	N	↑	↑	N	↑	↑	↑	N	↑	↓ then normalization few days later	NP			

^1^*p* < 0.01 as compared with healthy subjects; ^2^ No difference as compared with ICU patients with LT ≤ 393 s (*p* > 0.05); ^3^ *p* < 0.01 as compared with ICU patients with LT ≤ 393 s; ^4^ *p* < 0.05 as compared with ICU patients with LT ≤ 393 s. Abbreviations: ICU: Intensive care unit (adults); RRT: Renal replacement therapy; tPA: tissue plasminogen activator; N: Result within the reference range; ↑: Result above the reference range; ↓: Result below the reference range; NP: Not provided; NA: Not assessed.

**Table 11:** Conclusions of the previously published reviews.

First Author (VET Devices)	Type of the Review	Aim of the Review	Number and Type of Studies Included	Conclusions of the Authors
Görlinger et al. [69] (ROTEM, TEG and Quantra)	Narrative review	Review of coagulation abnormalities and inflammatory response associated with COVID-19, as well as highlight of what we still do not know about COVID-19 associated coagulopathy	8 studies (5 prospective, 3 retrospective)	VETs can detect the presence of hypercoagulability in critically ill COVID-19 patients, but further studies are needed to define the role of viscoelastometric testing in the management of patients VETs can be used to assess fibrinogen levels of COVID-19 patients receiving direct thrombin inhibitors (such as argatroban and bivalirudin) through functional fibrinogen measurement
Tsantes et al. [70] (ROTEM, TEG and Quantra)	Narrative review	Evaluation of the usefulness of VETs in clinical practice to guide anticoagulant treatments or predict prognosis	13 studies (8 prospective, 5 retrospective)	VETs can detect the presence of hypercoagulability in critically ill COVID-19 patients, but further studies are needed to establish reference ranges for each viscoelastic test, to define the common cut-off values of hypo- and hypercoagulability or threshold values to predict prognosis, or to guide anticoagulant, antiplatelet or fibrinolytic therapy
Hartmann et al. [71] (TEG)	Systematic review	Evaluation of the usefulness of TEG in clinical practice to identify and manage hypercoagulation associated with COVID-19	15 studies (5 prospective, 9 retrospective and one case report)	TEG can detect a hypercoagulable state in patients with COVID-19, and provides differential diagnostic insights alongside the ability to risk-stratify patients at elevated risk for complications such as VTE or kidney failure Further studies are needed to elucidate the optimal use of TEG to maximize patient benefit
Słomka et al. [72] (ROTEM and TEG)	Systematic review	Evaluation of the performance of TEG and TEM in the assessment of blood coagulation and fibrinolysis in patients with COVID-19	10 studies (2 prospective, 8 retrospective)	VETs can detect a hypercoagulable state and fibrinolysis shutdown in COVID-19 patients, and might be used to identify patients with high prothrombotic risk for whom an antithrombotic therapy would be benefic

## Data Availability

Data can be provided by contacting the authors.

## Supplementary Materials

The following are available online at https://www.mdpi.com/article/10.3390/jcm10081740/s1, Data S1: Search strategy, Data S2: PRISMA summary table, Data S3: Quality assessment of the retrieved studies.

## Appendix A. ROTEM® Reagents and Parameters

**Table A1:** ROTEM® reagents.

Assay	Reagent	Description	Heparin Neutralization
INTEM	Ellagic acid	Intrinsic pathway screening test	No
HEPTEM	Ellagic acid + Heparinase	Intrinsic pathway screening test with heparinase	Yes ^1^
EXTEM	Tissue factor + Polybrene	Rapid overview of the coagulation process	Yes ^2^
APTEM	Tissue factor + Aprotinin + Polybrene	Exploration of the fibrinolysis by comparison with the EXTEM results	Yes ^2^
FIBTEM	Tissue factor + Cytochalasin D + Polybrene	Functional detection of the fibrinogen level after platelet inhibition by cytochalasin D	Yes ^2^

^1^ Up to 7 IU/mL according to the manufacturer; ^2^ Up to 5 IU/mL according to the manufacturer.

**Table A2:** ROTEM® parameters.

Parameter	Description
CT (s)	Clotting time: time interval from the start of the run until a 2 mm clot forms
CFT (s)	Clot formation time: time interval from CT until a clot amplitude of 20 mm is reached
α angle (°)	Rate of clot formation
A(x) (mm)	Amplitude of the oscillation due to clotting x minutes after CT
MCF (mm)	Maximum clot firmness: maximum clot amplitude
LI(x) (%)	Clot lysis index: ratio between MCF and amplitude of the clot x minutes after CT
ML (%)	Maximum lysis: maximum fibrinolysis detected during the observation period, expressed as a percentage of MCF

## Appendix B. TEG® Reagents and Parameters (Haemonetics Corporation, Boston, MA, USA)

**Table A3:** TEG® reagents.

Assay	Reagents for TEG5000	Reagents for TEG6s	Description	Heparin Neutralization
RapidTEG (CRT)	Tissue factor + Kaolin + Heparinase if heparinase cups are used	Tissue factor + Kaolin	Rapid overview of the coagulation process	Yes (if heparinase cups were used for TEG5000), otherwise no
Kaolin TEG (CK)	Kaolin	Kaolin	Intrinsic pathway screening test	No
Kaolin TEG with heparinase (CKH)	Kaolin + Heparinase (heparinase cup)	Kaolin + Heparinase	Intrinsic pathway screening test with heparinase	Yes
TEG Functional Fibrinogen (CFF)	Tissue factor + Abciximab + Heparinase if heparinase cups are used	Tissue factor + Abciximab	Functional detection of the fibrinogen level after platelet inhibition by abciximab	Yes (if heparinase cups were used for TEG5000), otherwise no

**Table A4:** TEG® parameters.

Parameter	Description
R (min)	Reaction time: time to initial fibrin formation
K (min)	Kinetics time: time to clot formation
α angle (°)	Rate of clot formation
MA (mm)	Maximum amplitude: absolute clot strength
LY30 (%)	Fibrinolytic activity 30 min after maximum amplitude was reached

## Appendix C. Quantra® Reagents and Parameters (HemoSonics, LLC, Charlottesville, VA, USA)

**Table A5:** Quantra® QPlus cartridge and parameters.

Parameter	Reagents	Description	Heparin Neutralization	Manufacturer’s Reference Range
CT (s)	Kaolin (channel 1)	Clotting time after addition of kaolin	No	113–164 s
CTH (s)	Kaolin + Heparinase (channel 2)	Clotting time with heparinase after addition of kaolin	Yes	103–153 s
CT/CTH	None, calculated as the ratio of CT (channel 1) over CTH (channel 2)	Clot time ratio	NA ^1^	<1.4
CS (hPA)	Thromboplastin + Polybrene (channel 3)	Clot stiffness	Yes	13–33.2 hPa
FCS (hPA)	Thromboplastin + Abciximab + Polybrene (channel 4)	Fibrinogen contribution to overall clot stiffness after platelet inhibition with abciximab	Yes	1–3.7 hPa
PCS (hPA)	None, calculated as the difference between CS (channel 3) and FCS (channel 4)	Platelet contribution to clot stiffness	Yes	11.9–29.8 hPa

^1^ NA: not applicable.

**Table A6:** Quantra® QStat cartridge and parameters.

Parameter	Reagents	Description	Heparin Neutralization	Manufacturer’s Reference Range
CT (s)	Kaolin	Clotting time after addition of kaolin	No	113–164 s
CS (hPA)	Thromboplastin + Polybrene	Clot stiffness	Yes	13–33.2 hPa
CSL (%)	None, calculated as the normalized difference between the clot stiffness change after maximum clot stiffness in the absence of tranexamic acid and the corresponding clot stiffness change in the presence of tranexamic acid	Clot stability to lysis	Yes	93–100%
FCS (hPA)	Thromboplastin + Abciximab + Polybrene	Fibrinogen contribution to overall clot stiffness after platelet inhibition with abciximab	Yes	1–3.7 hPa
PCS (hPA)	None, calculated as the difference between CS and FCS	Platelet contribution to clot stiffness	Yes	11.9–29.8 hPa

## Appendix D. ClotPro® Reagents and Parameters (enicor GmbH, Munich, Germany)

**Table A7:** ClotPro® reagents.

Assay	Reagent	Description	Heparin Neutralization
IN-test	Ellagic acid	Intrinsic pathway screening test	No
HI-test	Ellagic acid + Heparinase	Intrinsic pathway screening test with heparinase	Yes
EX-test	Recombinant tissue factor + Polybrene	Rapid overview of the coagulation process	Yes
AP-test	Tissue factor + Aprotinin + Polybrene	Exploration of the fibrinolysis by comparison with the EX-test results	Yes
tPA-test	Recombinant tissue factor + Recombinant tPA + Polybrene	Exploration of the fibrinolysis by comparison with the EX-test results	Yes
FIB-test	Recombinant tissue factor + Cytolochalasin D + Abciximab + Polybrene	Functional detection of the fibrinogen level after dual platelet inhibition by cytochalasin D and abciximab	Yes
RVV-test	Reagent derived from Russell viper venom	Detection of factor Xa inhibitors (LMWH, DOAC)	No
ECA-test	Ecarin + Polybrene	Detection of direct thrombin antagonists	Yes
NA-test	None	Non-activated test for the exploration of non-activated coagulation in citrated blood	No

Abbreviations: LMWH: Low molecular weight heparin; UFH: unfractionated heparin; DOAC: Direct oral anticoagulant.

**Table A8:** ClotPro® parameters.

Parameter	Description
CT (s)	Clotting time: time interval from the start of the run until a 2 mm amplitude of oscillations due to clotting was reached
CFT (s)	Clot formation time: time interval from CT until a clot amplitude of 20 mm is reached
A(x) (mm)	Amplitude of the oscillation due to clotting x minutes after CT
MCF (mm)	Maximum clot firmness: maximum clot amplitude
ML (%)	Maximum lysis: maximum fibrinolysis detected during the observation period, expressed as a percentage of MCF
