# Supplementary material for: Viscoelastometric Testing to Assess Hemostasis of COVID-19: A Systematic Review
Source: J Clin Med. 2021 Apr 16;10(8):1740. doi: 10.3390/jcm10081740 (PMC8072929; doi:10.3390/jcm10081740)
Supplement: Supplementary file 1 [file jcm-10-01740-s001.zip › jcm-1179605-suppl.pdf]

Journal of
Clinical Medicine

MDPI

Supplementary materials

Viscoelastic testing to assess hemostasis of COVID-19: A systematic review

Marion Bareille [1,*], Michaël Hardy [2], Jonathan Douxfils [3,4], Stéphanie Roullet [5,6], Dominique Lasne [7], Jerrold H. Levy [8], Alain Stépanian [9], Sophie Susen [10], Corinne Frère [11], Thomas Lecompte [12] and François Mullier [1]

1 Namur Thrombosis and Hemostasis Center (NTHC), CHU UCL Namur, Université catholique de Louvain, 5530 Yvoir, Belgium; francois.mullier@uclouvain.be
2 Service d'anesthésiologie, CHU UCL Namur, Université catholique de Louvain, 5530 Yvoir, Belgium; michael.hardy@uclouvain.be
3 Namur Thrombosis and Hemostasis Center (NTHC), Département de pharmacie, Université de Namur, 5000 Namur, Belgium; jonathan.douxfils@unamur.be
4 Qualiblood S.A., 5000 Namur, Belgium
5 CHU Bordeaux, Service d'Anesthésie-Réanimation Tripode, 33000 Bordeaux, France
6 Biologie des maladies cardiovasculaire, University Bordeaux, INSERM U1034, 33600 Pessac, France; stephanie.roullet@chu-bordeaux.fr
7 Laboratoire d'hématologie générale, Hôpital universitaire Necker-Enfants Malades, AP-HP, 75015 Paris, France; dominique.lasne@aphp.fr
8 Departments of Anesthesiology, Critical Care, and Surgery (Cardiothoracic), Duke University School of Medicine, Durham, NC 27710, USA; jerrold.levy@duke.edu
9 Hôpital Lariboisière, Service d'Hématologie biologique, Institut de Recherche Saint-Louis, Université de Paris, AP-HP Nord-Université de Paris, EA 3518, 75010 Paris, France; alain.stepanian@aphp.fr
10 Laboratoire d'Hématologie-Hémostase, Université de Lille, CHU Lille, 59037 Lille, France; sophie.susen@chru-lille.fr
11 Department of Hematology, Pitié-Salpêtrière Hospital, Assistance Publique Hôpitaux de Paris, INSERM UMRS_1166, Sorbonne Université, 75013 Paris, France; corinne.frere@aphp.fr
12 Départements de Médecine, service d'angiologie et d'hémostase et Faculté de Médecine Geneva Platelet Group (GpG), Université de Genève et Hôpitaux Universitaires de Genève, 1205 Genève, Switzerland; thomaspierre.lecompte@hcuge.ch
* Correspondence: marion.bareille@uclouvain.be

Citation: Bareille, M.; Hardy, M.; Douxfils, J.; Roullet, S.; Lasne, D.; Levy, J.H.; Stépanian, A.; Susen, S.; Frère, C.; Lecompte, T.; Mullier F. Viscoelastometric Testing to Assess Hemostasis of COVID-19: A Systematic Review. *J. Clin. Med.* **2021**, *10*, 1740. https://doi.org/10.3390/jcm10081740

Received: 26 March 2021
Accepted: 12 April 2021
Published: 16 April 2021

Academic Editor: Angelo Claudio Molinari

Publisher's Note: MDPI stays neutral with regard to jurisdictional claims in published maps and institutional affiliations.

Copyright: © 2021 by the authors. Submitted for possible open access publication under the terms and conditions of the Creative Commons Attribution (CC BY) license (http://creativecommons.org/licenses/by/4.0/).

Data S1. Search strategy

S1.1. Pubmed database (until December 31st, 2020)

S1.1.1. Search

((((((((viscoelastic test) OR (thromboelastometry) OR (thromboelastography) OR (sonorheometry) OR (rotem) OR (teg) OR (quantra) OR (clotpro))))))) AND ((((covid 19) OR (coronavirus disease 2019) OR (severe acute respiratory syndrome coronavirus 2) OR (SARS-CoV-2))))) Filters: **from 1000/1/1 - 2020/12/31**

((("viscoelastic"[All Fields] OR "viscoelastically"[All Fields] OR "viscoelasticities"[All Fields] OR "viscoelasticity"[All Fields] OR "viscoelastics"[All Fields]) AND ("research design"[MeSH Terms] OR ("research"[All Fields] AND "design"[All Fields]) OR "research design"[All Fields] OR "test"[All Fields])) OR ("thrombelastography"[MeSH Terms] OR "thrombelastography"[All Fields] OR "thromboelastometry"[All Fields]) OR ("thrombelastography"[MeSH Terms] OR "thrombelastography"[All Fields] OR "thromboelastography"[All Fields]) OR "sonorheometry"[All

Fields] OR "rotem"[All Fields] OR "teg"[All Fields] OR "quantra"[All Fields] OR "clotpro"[All Fields]) AND ("covid 19"[All Fields] OR "covid 19"[MeSH Terms] OR "covid 19 vaccines"[All Fields] OR "covid 19 vaccines"[MeSH Terms] OR "covid 19 serotherapy"[All Fields] OR "covid 19 serotherapy"[Supplementary Concept] OR "covid 19 nucleic acid testing"[All Fields] OR "covid 19 nucleic acid testing"[MeSH Terms] OR "covid 19 serological testing"[All Fields] OR "covid 19 serological testing"[MeSH Terms] OR "covid 19 testing"[All Fields] OR "covid 19 testing"[MeSH Terms] OR "sars cov 2"[All Fields] OR "sars cov 2"[MeSH Terms] OR "severe acute respiratory syndrome coronavirus 2"[All Fields] OR "ncov"[All Fields] OR "2019 ncov"[All Fields] OR (("coronavirus"[MeSH Terms] OR "coronavirus"[All Fields] OR "cov"[All Fields]) AND 2019/11/01:3000/12/31[Date - Publication]) OR ("covid 19"[MeSH Terms] OR "covid 19"[All Fields] OR "coronavirus disease 2019"[All Fields]) OR ("sars cov 2"[MeSH Terms] OR "sars cov 2"[All Fields] OR "severe acute respiratory syndrome coronavirus 2"[All Fields]) OR ("sars cov 2"[MeSH Terms] OR "sars cov 2"[All Fields] OR "sars cov 2"[All Fields]))

Translations

viscoelastic: "viscoelastic"[All Fields] OR "viscoelastically"[All Fields] OR "viscoelasticities"[All Fields] OR "viscoelasticity"[All Fields] OR "viscoelastics"[All Fields]

test: "research design"[MeSH Terms] OR ("research"[All Fields] AND "design"[All Fields]) OR "research design"[All Fields] OR "test"[All Fields]

thromboelastometry: "thrombelastography"[MeSH Terms] OR "thrombelastography"[All Fields] OR "thromboelastometry"[All Fields]

thromboelastography: "thrombelastography"[MeSH Terms] OR "thrombelastography"[All Fields] OR "thromboelastography"[All Fields]

covid 19: ("COVID-19" OR "COVID-19"[MeSH Terms] OR "COVID-19 Vaccines" OR "COVID-19 Vaccines"[MeSH Terms] OR "COVID-19 serotherapy" OR "COVID-19 serotherapy"[Supplementary Concept] OR "COVID-19 Nucleic Acid Testing" OR "covid-19 nucleic acid testing"[MeSH Terms] OR "COVID-19 Serological Testing" OR "covid-19 serological testing"[MeSH Terms] OR "COVID-19 Testing" OR "covid-19 testing"[MeSH Terms] OR "SARS-CoV-2" OR "sars-cov-2"[MeSH Terms] OR "Severe Acute Respiratory Syndrome Coronavirus 2" OR "NCOV" OR "2019 NCOV" OR (("coronavirus"[MeSH Terms] OR "coronavirus" OR "COV") AND 2019/11/01[PDAT] : 3000/12/31[PDAT]))

coronavirus disease 2019: "covid-19"[MeSH Terms] OR "covid-19"[All Fields] OR "coronavirus disease 2019"[All Fields]

severe acute respiratory syndrome coronavirus 2: "sars-cov-2"[MeSH Terms] OR "sars-cov-2"[All Fields] OR "severe acute respiratory syndrome coronavirus 2"[All Fields]

SARS-CoV-2: "sars-cov-2"[MeSH Terms] OR "sars-cov-2"[All Fields] OR "sars cov 2"[All Fields]

S1.1.2. Results

63 articles

S1.2. Scopus database (until December 31st, 2020)

S1.2.1. Search

TITLE-ABS-KEY ((((((((viscoelastic AND test)
OR (thromboelastometry) OR (thromboelastography) OR (sonorheometry) OR (rotem) OR (teg) O
R (quantra) OR (clotpro)))))))) AND ((((covid 19) OR (coronavirus AND disease 2019) OR (severe
AND acute
AND respiratory AND syndrome AND coronavirus 2) OR (sars-cov-2))))) AND PUBYEAR < 2021

S1.2.2. Results

75 articles

Journal of *Clinical Medicine*

Data S2. PRISMA summary table

Section and Topic	Item #	Checklist item	Reported on page #
**TITLE**			
Title	1	Identify the report as a systematic review, meta-analysis, or both.	1
**ABSTRACT**			
Structured summary	2	Provide a structured summary including, as applicable: background; objectives; data sources; study eligibility criteria, participants, and interventions; study appraisal and synthesis methods; results; limitations; conclusions and implications of key findings; systematic review registration number.	1
**INTRODUCTION**			
Rationale	3	Describe the rationale for the review in the context of what is already known.	2
Objectives	4	Provide an explicit statement of questions being addressed with reference to participants, interventions, comparisons, outcomes, and study design (PICOS).	2
**METHODS**			
Protocol and registration	5	Indicate if a review protocol exists, if and where it can be accessed (e.g., Web address), and, if available, provide registration information including registration number.	NA
Eligibility criteria	6	Specify study characteristics (e.g., PICOS, length of follow-up) and report characteristics (e.g., years considered, language, publication status) used as criteria for eligibility, giving rationale.	3 + Table 1
Information sources	7	Describe all information sources (e.g., databases with dates of coverage, contact with study authors to identify additional studies) in the search and date last searched.	2-3
Search strategy	8	Present full electronic search strategy for at least one database, including any limits used, such that it could be repeated.	Supplementary material S1
Study selection	9	State the process for selecting studies (i.e., screening, eligibility, included in systematic review, and, if applicable, included in the meta-analysis).	3 + Table 1
Data collection process	10	Describe method of data extraction from reports (e.g., piloted forms, independently, in duplicate) and any processes for obtaining and confirming data from investigators.	3-4
Data items	11	List and define all variables for which data were sought (e.g., PICOS, funding sources) and any assumptions and simplifications made.	3-4
Risk of bias in individual studies	12	Describe methods used for assessing risk of bias of individual studies (including specification of whether this was done at the study or outcome level), and how this information is to be used in any data synthesis.	7 + Suplementary material S3
Summary measures	13	State the principal summary measures (e.g., risk ratio, difference in means).	NA

Synthesis of results	14	Describe the methods of handling data and combining results of studies, if done, including measures of consistency (e.g., I2) for each meta- analysis.	3-4
Risk of bias across studies	15	Specify any assessment of risk of bias that may affect the cumulative evidence (e.g., publication bias, selective reporting within studies).	7 + Supplementary material S3
Additional analyses	16	Describe methods of additional analyses (e.g., sensitivity or subgroup analyses, meta-regression), if done, indicating which were prespecified.	NA
**RESULTS**			
Study selection	17	Give numbers of studies screened, assessed for eligibility, and included in the review, with reasons for exclusions at each stage, ideally with a flow diagram.	5-6 + Figure 1
Study characteristics	18	For each study, present characteristics for which data were extracted (e.g., study size, PICOS, follow-up period) and provide the citations.	7-22 + Table 3 + Table 4
Risk of bias within studies	19	Present data on risk of bias of each study and, if available, any outcome level assessment (see item 12).	7 + Supplementary material S3
Results of individual studies	20	For all outcomes considered (benefits or harms), present, for each study: (a) simple summary data for each intervention group (b) effect estimates and confidence intervals, ideally with a forest plot.	22-58 + Table 5 + Table 6 + Table 7 + Table 8 + Table 9 + Table 10
Synthesis of results	21	Present results of each meta-analysis done, including confidence intervals and measures of consistency.	NA
Risk of bias across studies	22	Present results of any assessment of risk of bias across studies (see Item 15).	7 + Supplementary material S3
Additional analysis	23	Give results of additional analyses, if done (e.g., sensitivity or subgroup analyses, meta-regression [see Item 16]).	NA
**DISCUSSION**			
Summary of evidence	24	Summarize the main findings including the strength of evidence for each main outcome; consider their relevance to key groups (e.g., healthcare providers, users, and policy makers).	58-65
Limitations	25	Discuss limitations at study and outcome level (e.g., risk of bias), and at review-level (e.g., incomplete retrieval of identified research, reporting bias).	58-65
Conclusions	26	Provide a general interpretation of the results in the context of other evidence, and implications for future research.	58-65
**FUNDING**			
Funding	27	Describe sources of funding for the systematic review and other support (e.g., supply of data); role of funders for the systematic review.	66

Data S3. Quality assessment of the retrieved studies

First author (Country)	Device	Design	SIGN grade
**Iwasaki et al. (Japan) [26]**	ROTEM (NS)	Case report	3
**Pavoni et al. (Italy) [27]**	ROTEM gamma	Retrospective observational study	2-
**Boscolo et al. (Italy) [28]**	ROTEM delta	Prospective observational study	2+
**Corrêa et al. (Brazil) [29]**	ROTEM delta	Prospective observational study	2+
**Madathil et al. (USA) [30]**	ROTEM delta	Prospective observational study	2-
**Spiezia et al. (Italy) [31]**	ROTEM delta	Prospective observational case control study	2-
**Tsantes et al. (Greece) [32]**	ROTEM delta	Prospective observational study	2-
**Al-Ghafry et al. (USA) [33]**	ROTEM delta	Retrospective observational study	2-
**Creel-Bulos et al. (USA) [34]**	ROTEM delta	Retrospective observational study	2+
**Hoechter et al. (Germany) [35]**	ROTEM delta	Retrospective observational case control study	2+
**Roh et al. (USA) [36]**	ROTEM delta	Retrospective observational case control study	2-
**Kong et al. (United Kingdom) [37]**	ROTEM delta	Case report	3
**Raval et al. (USA) [38]**	ROTEM delta	Case report	3
**Nougier et al. (France) [39]**	Modified ROTEM delta (TEM-tPA)	Prospective observational case control study	2-
**Weiss et al. (France) [40]**	Modified ROTEM delta (TEM-tPA)	Prospective observational case control study	2-
**Almskog et al. (Sweden) [41]**	ROTEM sigma	Prospective observational study	2-
**Collett et al. (Australia) [42]**	ROTEM sigma	Prospective observational study	2-
**Ibañez et al. (Spain) [43]**	ROTEM sigma	Prospective observational study	2-
**Kruse et al. (Germany) [44]**	ROTEM sigma	Prospective observational study	2+
**Pavoni et al. (Italy) [45]**	ROTEM sigma	Prospective case controls observational study	2-
**Spiezia et al. (Italy) [46]**	ROTEM sigma	Prospective case controls observational study	2-
**Van der Linden et al. (Sweden) [47]**	ROTEM sigma	Cross-sectional cohorts study	2-
**Blasi et al. (Spain) [48]**	ROTEM sigma	Retrospective observational study	2-
**Van Veenendaal et al. (The Netherlands) [49]**	ROTEM sigma	Retrospective observational study	2+
**Lazar et al. (USA) [50]**	ROTEM sigma	Case report	3
**Wright et al. (USA) [51]**	TEG (NS)	Retrospective observational study	2+
**Panigada et al. (Italy) [52]**	TEG5000	Prospective observational study	2-
**Cordier et al.**	TEG5000	Retrospective observational study	2+

**(France) [53]**			
**Hightower et al. (USA) [54]**	TEG5000	Retrospective observational study	2-
**Maatman et al. (USA) [55]**	TEG5000	Retrospective multi-center observational study	2+
**Mortus et al. (USA) [56]**	TEG5000	Retrospective cohort study	2+
**Sadd et al. (USA) [57]**	TEG5000	Retrospective observational cohort study	2-
**Yuriditsky et al. (USA) [58]**	TEG5000	Retrospective observational study	2+
**Bocci et al. (Italy) [59]**	TEG6s	Prospective observational study	2+
**Stattin et al. (Sweden) [60]**	TEG6s	Prospective observational study	2-
**Vlot et al. (The Netherlands) [61]**	TEG6s	Prospective observational study	2-
**Patel et al. (United Kingdom) [62]**	TEG6s	Retrospective observational study	2-
**Salem et al. (United Arab Emirates) [63]**	TEG6s	Retrospective observational study	2+
**Shah et al. (United Kingdom) [64]**	TEG6s	Multicenter retrospective observational study	2+
**Fan et al. (Singapore) [65]**	TEG6s	Case report	3
**Masi et al. (France) [66]**	Quantra	Prospective single-center cohort study	2-
**Ranucci et al. (Italy) [67]**	Quantra	Prospective observational study	2-
**Bachler et al. (Austria) [24]**	ClotPro	Retrospective study	2+
**Zátroch et al. (Hungary) [68]**	ClotPro	Case report	3
